# Supplementary material for: Development of an imputation model to recalibrate birth weights measured in the early neonatal period to time at delivery and assessment of its impact on size-for-gestational age and low birthweight prevalence estimates: a secondary analysis of a pregnancy cohort in rural Nepal
Source: BMJ Open. 2022 Jul 12;12(7):e060105. doi: 10.1136/bmjopen-2021-060105 (PMC9277385; doi:10.1136/bmjopen-2021-060105)
Supplement: Supplementary data [file bmjopen-2021-060105supp001.pdf]

Annexes:

Table A1: Regression outputs for the training model, chlorhexidine trial substudy (n= 4148 weight measurements on 456 infants) and the analytical model, NOMS trial (n=28016 infants, 27836 pregnancies and 21770 women) with complete data on the covariates.

	CHX<br>*Weight, grams*<br>*(standard error)*	*NOMS*<br>*Weight, grams*<br>*(standard error)*
Parity<br>(reference=previous birth)	-163.7 (49.6)	-202.4<br>6.0
Sex<br>(reference=female)	50.6 (37.7)	110.3<br>4.7
Gestational age in weeks, centered at 40 weeks	52.7 (7.9)	31.5<br>0.8
Neonatal death<br>(reference = survives neonatal period or lost-to-follow-up)	N/A	-323.9<br>19.8
Maternal education in years	-0.5 (3.3)	13.8<br>0.7
Maternal age in years	10.7 (6.0)	5.4<br>0.6
Multiple gestation (twins or triplets)<br>(reference = singleton)	N/A	-658.5<br>23.8
Intercept	2745.1 (90.0)	

N/A: there were no twins/triplets or neonatal deaths in the first 10 days in the CHX substudy.

Table A2: Akaike's *An Information Criteria* (AIC) for five models of predicted weight at delivery (training model), chlorhexidine trial (n= 4148 weight measurements on 456 infants)

Model	Covariates	Degrees freedom	AIC
Full model	Baby gender, Gestational age at delivery, Parity, Maternal age, and Maternal years of education,	13	48369.92
Model 1	Excluding Parity	12	48388.18
Model 2	Exclude Education	12	48376.52
Model 3	Exclude Education and Maternal age	11	48378.86
Model 4	Exclude: Education, Maternal age, and Parity	10	48396.61

AIC: Akaike's *An Information Criteria*

Table A3: Proportion of infants with weight missing or weight measured greater than 72 hours post-delivery stratified by preterm birth, survival status, and maternal parity and education, NOMS data

	Type of missingness % (95% CI)	
**Proportion missing with:**	**Weight missing**	**Weight measured greater than 72 hours after delivery**
Preterm birth (<37 weeks)	16.3 (15.3 – 17.4)	13.6 (12.6 – 14.7)
Non-preterm birth (>=37 weeks)	11.7 (11.3 – 12.1)	13.5 (13.1 – 14.0)
Infant alive at end of study	10.6 (10.3 – 11.0)	13.6 (13.2 – 14.1)
Infant deceased at end of study	61.7 (58.7 – 64.6)	4.8 (3.1 – 7.4)
No previous births	15.7 (15.0 – 16.4)	17.0 (16.2 – 17.8)
Any previous births	10.9 (10.5 – 11.3)	12.0 (11.5 – 12.5)
No education	11.2 (10.8 – 11.7)	11.6 (11.2 – 12.1)
Any education	14.9 (14.2 – 15.6)	17.6 16.8 – 18.4)

CI: confidence intervals
