# Supplementary material for: Development of an imputation model to recalibrate birth weights measured in the early neonatal period to time at delivery and assessment of its impact on size-for-gestational age and low birthweight prevalence estimates: a secondary analysis of a pregnancy cohort in rural Nepal
Source: BMJ Open. 2022 Jul 12;12(7):e060105. doi: 10.1136/bmjopen-2021-060105 (PMC9277385; doi:10.1136/bmjopen-2021-060105)
Supplement: Author's manuscript [file bmjopen-2021-060105.draft_revisions.pdf]

BMJ Open

BMJ Open is committed to open peer review. As part of this commitment we make the peer review history of every article we publish publicly available.

When an article is published we post the peer reviewers' comments and the authors' responses online. We also post the versions of the paper that were used during peer review. These are the versions that the peer review comments apply to.

The versions of the paper that follow are the versions that were submitted during the peer review process. They are not the versions of record or the final published versions. They should not be cited or distributed as the published version of this manuscript.

BMJ Open is an open access journal and the full, final, typeset and author-corrected version of record of the manuscript is available on our site with no access controls, subscription charges or pay-per-view fees (http://bmjopen.bmj.com).

If you have any questions on BMJ Open's open peer review process please email info.bmjopen@bmj.com

BMJ Open

Birthweight imputation and the impact on small-for-gestational age and low birthweight estimates in Nepal.

Journal:	*BMJ Open*
Manuscript ID	bmjopen-2021-060105
Article Type:	Original research
Date Submitted by the Author:	15-Dec-2021

Complete List of Authors:	Hazel , E; Johns Hopkins University Bloomberg School of Public Health,<br>Mullany, Luke C.; Johns Hopkins University Bloomberg School of Public Health, International Health<br>Zeger, Scott ; Johns Hopkins University Bloomberg School of Public Health<br>Mohan, Diwakar; Johns Hopkins University Bloomberg School of Public Health<br>Subedi, Seema; Johns Hopkins University Bloomberg School of Public Health, International Health<br>Tielsch, James; George Washington University School of Public Health and Health Services, Global Health<br>Khatry, Subarna; Nepal Nutr Intervent Project Sarlahi<br>Katz, Joanne; Johns Hopkins University Bloomberg School of Public Health, International Health

Keywords:	PUBLIC HEALTH, Maternal medicine < OBSTETRICS, NEONATOLOGY

SCHOLARONE™
Manuscripts

I, the Submitting Author has the right to grant and does grant on behalf of all authors of the Work (as defined in the below author licence), an exclusive licence and/or a non-exclusive licence for contributions from authors who are: i) UK Crown employees; ii) where BMJ has agreed a CC-BY licence shall apply, and/or iii) in accordance with the terms applicable for US Federal Government officers or employees acting as part of their official duties; on a worldwide, perpetual, irrevocable, royalty-free basis to BMJ Publishing Group Ltd ("BMJ") its licensees and where the relevant Journal is co-owned by BMJ to the co-owners of the Journal, to publish the Work in this journal and any other BMJ products and to exploit all rights, as set out in our licence.

The Submitting Author accepts and understands that any supply made under these terms is made by BMJ to the Submitting Author unless you are acting as an employee on behalf of your employer or a postgraduate student of an affiliated institution which is paying any applicable article publishing charge ("APC") for Open Access articles. Where the Submitting Author wishes to make the Work available on an Open Access basis (and intends to pay the relevant APC), the terms of reuse of such Open Access shall be governed by a Creative Commons licence – details of these licences and which Creative Commons licence will apply to this Work are set out in our licence referred to above.

Other than as permitted in any relevant BMJ Author's Self Archiving Policies, I confirm this Work has not been accepted for publication elsewhere, is not being considered for publication elsewhere and does not duplicate material already published. I confirm all authors consent to publication of this Work and authorise the granting of this licence.

For peer review only

Birthweight imputation and the impact on small-for-gestational age and low birthweight estimates in Nepal.

Elizabeth Hazel[a], PhD, Luke C. Mullany[a], PhD, Scott L. Zeger[a], PhD, Diwakar Mohan[a], DrPH MD,Seema Subedi[a], MPH, James M. Tielsch[b], PhD MHSc, Subarna K. Khatry[a,c], MBBS, Joanne Katz[a], ScD.

Affiliations: [a]Johns Hopkins Bloomberg School of Public Health, Baltimore, Maryland, USA; [b]George Washington University Milken Institute School of Public Health, Washington DC, USA; and [c]Nepal Nutrition Intervention Project-Sarlahi (NNIPS), Sarlahi, Nepal.

Address correspondence to: Elizabeth Hazel, Department of International Health, Johns Hopkins Bloomberg School of Public Health, 615 N. Wolfe St. Room W5504**,** Baltimore, MD 21205, USA, ehazel1@jhu.edu, +14106148821

Acknowledgements:

We would like to acknowledge Prof. Anne CC Lee at Harvard Medical School and Prof. Robert Black at Johns Hopkins Bloomberg School of Public Health for their technical contributions, and the study staff and participants at the Nepal Nutritional Intervention Project-Sarlahi site.

For peer review only

Abstract

Objectives: In low-income countries, birthweights for home deliveries are often measured after delivery, at the nadir when babies may lose up of 10% of their birthweight, overestimating the proportion of small-for-gestational (SGA). We aimed to develop an imputation model that predicts the "true" birthweight using weights measured in the days post-delivery.

Setting: This is a secondary analysis using pregnancy cohort data from two studies in Sarlahi district, Nepal.

Participants: The participants are (1) 457 neonates with longitudinal weight data from 2002-2005, a subsample of a larger clinical trial testing skin cleansing of babies with chlorhexidine (CHX); and (2) 30,680 neonates with weights measured throughout the early neonatal period from a study on neonatal skin massage (Nepal Oil Massage Study (NOMS)).

Primary and secondary outcome measures: We developed an empirical Bayes model of early neonatal weight change using CHX trial longitudinal data. We then applied this model to the NOMS dataset to impute a "true" weight at delivery.

The primary outcomes are the relative and absolute differences in SGA and low birth weight (<2500 grams) status comparing the imputed and measured birthweights. Secondary outcomes are the differences in large-for-gestational age and descriptive data on neonatal growth from the CHX data.

Results: In this sample, the proportion of SGA is reduced by 13% and low birthweight is reduced by 16% when using the imputed birthweights. The proportion of babies born large-for-gestational age increased by 30%. First births, multiples, reduced gestational age at delivery, poorer maternal

For peer review only - http://bmjopen.bmj.com/site/about/guidelines.xhtml

education, and babies that later died during the neonatal period were estimated to have a lower weight at delivery.

Conclusions: Using weights measured at or around the nadir over-estimates the prevalence of SGA and low birthweight. Studies in low-income settings with high levels of home births should consider a similar imputation model to generate more accurate population estimates of vulnerable newborns.

Article Summary

Strengths and limitations of this study

- The strengths of this study are that we developed our model of early neonatal growth using longitudinal data of daily weight change.
- Both data sets were sampled from a similar population of infants in rural Nepal, although there may be differences due to secular trends, the median birthweights and neonatal mortality rates of the two samples are similar.
- Both data sets included high quality gestational age and weight measurements taken on a population-representation sample with relatively low levels of missing data.
- The limitations of the study are that the birthweights are modelled and not empirical data.
- The model may not be generalizable to low-income settings outside of South Asia where prevalence of IUGR is lower.

For peer review only

Introduction

In 2010, 32.4 million babies in low-and-middle income countries (LMICs) were estimated to be small-for-gestational age (SGA), over a quarter of all live births and these babies had an 83% higher risk of dying the first month of life. [1,2] SGA is defined as a birthweight-for-gestational age less than the 10th percentile of a standard infant reference population and severe SGA is less that the 3rd percentile. A low weight at birth (defined as babies born less than 2500g regardless of gestational age) may result from a preterm birth (births less than 37 completed weeks gestational age), intra-uterine growth restriction (IUGR), or a combination of the two. The etiology of babies born with IUGR is likely distinct from those born preterm, and perhaps even different from babies born early with IUGR. To better understand the epidemiology of low birthweight babies, practitioners and researchers have moved to monitoring birthweight by gestational age, although low birthweight prevalences are still appropriate to use in setting where gestational age ascertained by ultra-sound is rare.[3] Some babies will be on the smaller end of the curve naturally, but in LMICs where SGA estimates are high, it can be used as a proxy for IUGR prevalence. SGA and low birthweight prevalence in LMICs are essential indicators of population health.

Along with gestational age, an accurate birthweight measured close to the time of birth is critical for estimating SGA. This is because neonatal weight decreases for the first 2-3 days after birth (known as the *nadir* or period of minimum neonatal weight), before starting to increase. A systematic review of breastfed, healthy, singleton babies from high income countries found the nadir of mean weight loss of 7-8% at day three. Many neonates in these studies had weight loss greater than 7% and up to 10% for exclusively breastfed babies.[4] Weights of babies taken during the nadir may bias SGA estimates, overestimating the prevalence.

For peer review only - http://bmjopen.bmj.com/site/about/guidelines.xhtml

For facility deliveries, birthweight is typically measured within the first six hours after delivery, reflecting the true weight at the time of birth. For babies born outside of facilities in LMICs, the risk of IUGR is higher, and SGA estimates based solely on facility births will underestimate the population-level prevalence. For babies born in the community, it may take 24 to 72 hours before a health worker can measure weight, often at the nadir of the infant's weight loss resulting in overestimation of LBW and SGA. Large-for-gestational age (LGA) or babies with a birthweight >90th centile have associated health risks.[5,6] Birthweights measured in the nadir may also under-estimate the true prevalence of LGA.

In this study, we aimed to generate estimates of SGA, LGA, and low birth weight using an imputation model that extrapolates infant weights measured post-delivery (around the nadir) to an imputed weight at the time of birth. In developing and applying the models, we used secondary data from community-based clinical trials in Nepal, a lower-middle income country in South Asia. The South Asia region has the highest prevalence of SGA in the world; a third of babies are born small (34%) compared to the global rate (19%).[7]

Methods

Data sources

We used two pregnancy cohort datasets from rural Nepal to build and test the model (table 1). The training dataset was derived from a cluster-randomized community-based trial examining the effect of chlorhexidine full body wipe at delivery and chlorhexidine umbilical stump application on neonatal omphalitis and neonatal mortality (CHX).[8,9] From 2002 to 2005, pregnant women were recruited approximately in the sixth month of pregnancy and infants were followed-up through the first 28 days of life. The dataset where we imputed weight at delivery was from another cluster-randomized community-based trial called the Nepal Oil Massage Study (NOMS),

For peer review only - http://bmjopen.bmj.com/site/about/guidelines.xhtml

(clinicaltrials.gov #NCT01177111). From 2010-2017, pregnant women were enrolled through community surveillance, and their newborns were randomized to receive postnatal massage using either sunflower or mustard seed oil to determine the impact of oil type on neonatal morbidity and mortality. Both studies took place in rural Sarlahi district in southern Nepal. In this area, less than half of the women delivery in health facilities (40%), c-sections are very low (<1%) and generally 90% of infants are breastfed within the first day of delivery.[10]

Table 1: Description of data sets used for analysis.

	Training data	Study data
Name	Chlorhexidine trial (CHX)	Oil massage trial (NOMS)
Setting	Sarlahi district, Nepal	Sarlahi district, Nepal
Years of data collection	2002-05	2010-17
Number of livebirths included in the analysis	457	30,680
Male gender (%)	57.6	51.7
Median gestational age (weeks)	39.7	39.4
Median birthweight (non-imputed, grams)	2706	2740
Median first weight measured (hours)	4.1	14.9
Pre-term, <37 weeks gestational age (%)	14.9	15.7
Number of neonatal deaths	0	1,031
Gestational age ascertainment	Last menstrual period	Last menstrual period
Instrument used to measure infant weight	Seca Digital Scale Model 727 and Tanita digital infant weight scale	Tanita digital infant weight scale

In the CHX trial, a subset of infants was selected for a longitudinal study of early neonatal weight change. Newborns with weight measured in the first six hours after delivery were systematically sampled for the longitudinal sub-study, and then followed for daily weight measurements through the first 10 days of life. A total of 457 infants from the CHX trial were selected for the sub-study (table 1), and the longitudinal weight measures from these infants were used to build a regression

For peer review only - http://bmjopen.bmj.com/site/about/guidelines.xhtml

model of weight change in the first 10 days of life. Gestational age was ascertained by timing of last menstrual period (LMP) before pregnancy. Recall of LMP was relatively short as it was ascertained during visits at five-week intervals for pregnancy surveillance of women of childbearing age. Multiples and neonates that did not survive were not included in the CHX subsample of longitudinal data.

For the NOMS study, women in the study area were visited approximately every five weeks and asked about the timing of their LMP. Pregnant women were enrolled throughout the pregnancy (median gestational age at enrollment was 14.1 weeks) and some women contributed multiple pregnancies to the study data. Birthweight was collected on all liveborn infants who survived at least until the first postnatal study team visit. Information on maternal age, parity, education, household wealth and infant sex and survival status were collected through interviews with the mother. In both studies, birthweight was measured at home by trained study staff using the same digital scales accurate to 10g. If a woman delivered at a facility, the baby's weight was measured when she returned home.

Descriptive analysis

For the CHX longitudinal data, we calculated the nadir weight, time in days to reach the nadir, and time in days to return to the birthweight in the 10-day period for the CHX study. To make these estimates, we developed a function that extends the predicted weights from time of delivery to a maximum observed time in units of days (within the 10-day period). The function then finds the time in days when the predicted weight achieves it minimum; and when the weight returns closest to the birth weight. We reported the median and interquartile range for these values and plotted probability of return to birthweight. Babies that did not return to their birthweight in the 10-day period were considered censored.

For peer review only - http://bmjopen.bmj.com/site/about/guidelines.xhtml

For the NOMS data, we described the sample and analyzed the pattern of missing weights and weights taken greater than 72 hours after delivery to determine if babies with missing and non-missing weights differed by preterm status (<37 weeks), survival status through the neonatal period, and mother's parity and educational level.

Predicting birthweight

Our analytic goal was to impute weight at birth in the NOMS dataset by estimating, then simulating from the conditional distribution of each child's birthweight (weight at t=0) given a single measurement at a known later time t>0 (case 1) or given missing weight (case 2).

In both cases, the strategy had three steps: (1) use the NOMS data to model the dependence of population average weight on age at measurement and other key predictors; (2) use the longitudinal CHX study to estimate the dependence of birthweight on later weights and assume this relationship holds approximately for the NOMS data as well; (3) integrate (1) and (2) to obtain the optimal linear predictor of birthweights in NOMS given the single NOMS measurement on each child at age t.

For step (1) using NOMS data, we regressed the observed weight (w_t) at age t in hours on: a smooth function of maternal age and education in years, the neonate's survival status by day of age, infant sex, an indicator of preterm birth, and whether the birth was a multiple or singleton, and whether it was the mother's first birth (parity). Neonatal survival status was defined as a liveborn death in the first 28 days of life with the reference being children who were alive through the end of the study (28 days after birth) or alive at the time they were lost-to-follow-up. We used this model to obtain the estimated weight (w_{it}^{NOMS}) for infant *i* at their measurement time *t* and their predicted birth weight (w_{i0}^{NOMS}) at *t*=0.

For peer review only - http://bmjopen.bmj.com/site/about/guidelines.xhtml

Using the CHX data, step (2) was to fit a linear mixed effect model with the repeated weights as the outcomes. The fixed effects included a natural cubic spline of infant's age (knots at day 2 and 4), parity, maternal education/age, infant sex, and gestational age at birth; random effects include the intercept and linear age. This model enabled us to estimate the conditional distribution of the unknown birthweight given one or more weights at later times, controlling for fixed effects. The mean of this distribution is the empirical Bayes estimate of birthweight. We reported the regression outputs for the CHX model by adjusted covariate. We applied the variance parameters from the training model to NOMS data to generate the distribution of the imputed birthweights for NOMS. In the final step (3), we applied the relationship between birthweight and subsequent weights derived in step (2) to the NOMS observed and predicted weights to obtain an improved prediction of the NOMS infant's birthweights. For the infants missing birthweight data, we predicted their birthweight by modeling the same fixed effects as previously mentioned using a random forest model. Infants with missing parity, survival status, maternal age/education, gestational age, singleton/multiples, or sex data were excluded from the imputation.

Comparing size-for-gestational age and low birth weight using raw and imputed weights

We generated appropriate for gestational age (AGA) (10th – 90th centiles), SGA (10th centile), and LGA (90th centile) using Intergrowth-21 standards extrapolated up to 22+0 to 44+6 weeks GA[1] (original model was 24+0 to 42+6 weeks). [11] We generated imputed estimates of AGA, SGA, LGA and low birth weight (< 2500g) prevalences with multiple imputation (m=5) in Stata 16.1. [12] We compared these prevalences using the imputed birthweight versus measured weight within 72 hours.

[1] Personal Communication, Eric Ohuma, London School of Hygiene and Tropical Medicine, September 2021

For peer review only - http://bmjopen.bmj.com/site/about/guidelines.xhtml

Ethical considerations

This is a secondary data analysis study of two trials for which institutional review board approvals were obtained from the Johns Hopkins Bloomberg School of Public Health (FWA00000287) and Nepal Health Research Council (FWA 00000957). All participants gave their informed consent to take part in both studies. This secondary data analysis was considered exempt by the Johns Hopkins Bloomberg School of Public Health institutional review board and conforms to the principles embodied in the Declaration of Helsinki.

Patient and Public Involvement

The patients and public were not involved in the design, conduct, reporting or dissemination of this research. Accurately measuring the health and nutritional status of infants is important to clinicians, researchers and families in Nepal, however, we did not seek the public's priorities, experience or preferences when designing this research question. We did not contact the original participants in the two studies used in this secondary data analysis.

Results

The median birthweight in the CHX trial (training data) was 2718 (IQR 2482 – 2981) (table 2). The median time of nadir was 2.1 days and the median weight at the nadir was 2607 (2773 – 2854); a relative weight loss of 2% or 55 grams. Median return to birthweight was 4.4 days (fig. 1). Eighty-four infants did not regain their birthweight with the 10 days.

Table 2: Neonatal growth during the first 10 days of life, chlorhexidine trial (n= 4148 weight measurements on 456 infants)

	Median (IQR)
Birthweight (grams)	2718 (2482 – 2982)
Nadir weight (grams)	2607 (2373 – 2854)
Time to reach nadir (days)	2.1

For peer review only - http://bmjopen.bmj.com/site/about/guidelines.xhtml

	(1.9 – 2.3)
Time to return to birthweight (days)	4.4<br>(3.7 – 6.0)
Number of babies that did not return to birthweight within the 10 days	84

IQR: Interquartile range

From the model outputs, we found babies born to mothers with no previous births were estimated to have a lower than average birthweight and baby girls born at a greater gestational age born to older mothers were estimated to have higher birthweights on average (Appendix table A1). There was minimal impact of maternal education on birthweight adjusted by other covariates.

There were 31,116 livebirths from 22,495 women in the NOMS dataset (table 3). We imputed a birthweight on 99% of the livebirths (n=30,996) with complete covariate data and gestational age data. Of those livebirths, 3,863 were missing a weight measurement and we were able to impute a birthweight for 98% (n=3,769). The pattern of missing birthweight was not random (appendix table A2). Infants without a measured weight were more likely to be born to nulliparous mothers, with more education. Infants who were preterm were more likely to have a missing birthweight (16% preterm compared to 12% full term) and over half of deceased infants did not have a weight measure (62%) compared to a tenth of infants who survived during the neonatal period (11%). Those infants with a weight measured after 72 hours showed a similar pattern.

Table 3: Description of covariate data for all live births, NOMS study

	**All live births (n=31,116) % (n)**
Maternal age at LMP (years)	
<18	15.5 (4,823)
18-35	82.3 (25,622)
>35	2.2 (671)
Missing	0
Maternal education	

For peer review only

For peer review only - http://bmjopen.bmj.com/site/about/guidelines.xhtml

No school	67.0 (20,855)
1-5 years	8.5 (2,638)
>5 years	24.4 (7,595)
Missing	0.1 (28)
Parity (live or stillbirth)	
No previous births	31.5 (9,794)
1	27.4 (8,531)
2	19.6 (6,108)
3	10.8 (3,370)
4+	10.1 (3,147)
Missing	0.5 (166)
Gestational age	
Term (≥37 - <45 weeks)	84.3 (26,243)
Moderate-to-Late preterm (≥32-<37 weeks)	13.7 (4,262)
Early preterm (≥28-<32 weeks)	1.6 (501)
Extremely preterm (<28 weeks)	0.4 (110)
Missing	0
Infant sex	
Female	48.0 (14,925)
Male	51.7 (16,099)
Missing	0.3 (92)
Multiple births	
Singleton	98.4 (30,616)
Twin/ Triplets	1.6 (500)
Missing	0
Infant survival status	
Alive at 28 days	96.6 (30,041)
Lost-to-follow-up	3.5 (1,076)
Deceased by 28 days	3.3 (1,031)
Missing	(0.1) 44
Missing birth weight	12.4 (3,863)
Timing of birthweight measurement (among non-missing birthweight)	
0-5 hours	17.5 (4,777)
6-23 hours	57.9 (15,773)
24-72 hours	11.1 (3,018)
>72 hours	13.5 (3,685)
Timing missing	0

For the NOMS imputation, at day 0 the imputed birthweights were at or slightly higher than the measured weights and on days 1-3 (the nadir) the imputed birthweight is even higher (fig. 2). During days 4-9 and 10+, when the baby begins to regain and surpass birthweight, the imputation

For peer review only - http://bmjopen.bmj.com/site/about/guidelines.xhtml

reduces the weights back down to hour=0 (fig. 2). On average, babies that did not survive the neonatal period had an imputed weight 368.6g less than babies that did, and multiples had an imputed weight 759.1g less than singleton babies. There was a small amount of heaping at 4500g for the measured weights; n=70 babies at exactly 4500g or a heaping index of 35% (+/- 250 grams) (fig. 2).

Using the imputed birthweights, we found differences in the SGA prevalence compared to weights taken within 72 hours after delivery (table 4). Using the imputed birthweight decreased the proportion of babies with low birthweight from 30% (95% CI: 30-31%) to 25% (95% CI: 25-26%). SGA prevalence using measured weights was 49% (95% CI: 48-49%) and 42% (95% CI: 42-43%) using imputed weights. The prevalence of LGA babies was similar in absolute terms (3.8% using the raw birthweights compared to 4.9% using the imputation). However, in relative terms, this is a 29% increase in the estimate of LGA from the measured to imputed.

Table 4: **Comparison of low birthweight, small-for-gestational age, and large-for-gestational age prevalences.**

	Measured weight <72hr % (95% CI)	Imputed birthweight % (95% CI)	Relative change (%)	Absolute difference (pp)
Low birth weight	30.1 (29.5 – 30.7)	25.4 (24.9 – 25.9)	15.6%	- 4.7
Appropriate for gestational age (90th - 10th centile)	47.7 (47.1 – 48.3)	52.7 (52.1 – 53.4)	10.5%	+5
Small-for-gestational age (<10th centile)	48.5 (47.9 – 49.1)	42.3 (41.7 – 42.9)	12.8%	- 6.2
Large-for-gestational age (>90th centile)	3.8 (3.6 – 4.1)	4.9 (4.6 – 5.2)	30%	+1.1
Sample size	23,568	30,996		

CI: confidence intervals; pp: percentage points

Discussion

For peer review only

For peer review only - http://bmjopen.bmj.com/site/about/guidelines.xhtml

Birthweights measured during the nadir overestimate the prevalence of SGA and LBW. The proportion of babies with SGA is reduced by a relative 13% and LBW by a relative 16% when including the birthweights imputed to time of delivery. Fonseca et al., modelled neonatal weight loss among full term, breastfed, singleton Portuguese infants with weight measured within 96 hours.[13] They fit several longitudinal models of weight change, but the goal was to examine the patterns and estimate a nadir, not to extrapolate the infant weights back to a "true" birthweight. This model was done in a high income setting with low SGA prevalence and different patterns of facility delivery/c-section compared to many LMICs in South Asia.

In the first week after delivery, there is evidence that infants follow a broadly consistent pattern of weight change. Weight loss two to three days postnatally is primarily due to loss of water weight – specifically extracellular volume, likely the process of re-adapting the cardio-pulmonary system for life outside the womb.[14] Even babies born preterm lose weight primarily due to reduced extracellular volume.[15,16] However, IUGR babies may have a different pattern of weight change. One study of SGA/term and SGA/preterm babies found weight loss was due primarily to catabolism rather than loss of extracellular volume.[17] Another study comparing SGA to appropriate for gestational age, preterm babies found less postnatal diuresis in the SGA babies.[18] In settings with a high prevalence of SGA, population level estimates of postnatal growth in the first week are likely to be different compared to low SGA prevalence areas – due to different growth patterns in babies with IUGR. This model should be tested in other settings but has the potential to be used for correction of birthweight measured in the days post-delivery, particularly in South Asian LMICs where SGA prevalence is high compared to other regions.

The main strength of this study is that we had a very large dataset with a relatively small proportion of missing covariates. In both the CHX and NOMS studies, we have high quality gestational age

For peer review only - http://bmjopen.bmj.com/site/about/guidelines.xhtml

at birth and infant weight data were taken on a population-representative sample of infants - born at home and in facilities. The longitudinal training and analytical data were collected in the same geographic area from the same study site, and data collection was managed by the same research organization, the Nepal Nutritional Intervention Study Sarlahi (NNIPS). Another strength of this study is we were able to use daily, longitudinal weights to build the model of early neonatal growth.

One limitation is the differences between the training and analytical datasets. Difference in timing of data collection: CHX (2002 – 2005) versus NOMS (2010-2017). Babies born during the NOMS data collection may have a different weight change pattern compared to babies born in CHX trial due to secular trends in maternal health and nutritional status. The percent of women delivering at home has declined from CHX to NOMS, primarily due to the cash incentive program in Nepal that pays women to deliver in a facility.[19] However, the median birthweights are similar and the neonatal mortality rates are similar as well (30.06 neonatal deaths per 1000 livebirths in CHX and 31.18 per 1000 live births in NOMS (personal communication, Joanne Katz, 16th November 2021)). [9]

All infants in the CHX subsample of longitudinal weights survived through the neonatal period and were singleton births. This is an important difference in the underlying population of the two samples (CHX versus NOMS) since multiples typically have lower birthweight and very SGA/preterm/low birth weight babies are more likely to die in the few days after delivery.

Another limitation is that the birthweights are not missing at random in NOMS. More than half the infants died before they could be measured, and more preterm babies did not have a weight measurement. We found infants of women with more education and wealth were missing birthweight, perhaps because they were more mobile and more likely to move out of the study area, or more likely to deliver at a facility and returned home after some days. Nulliparous women

For peer review only - http://bmjopen.bmj.com/site/about/guidelines.xhtml

were also more likely to have no weights measured on their infants. This may be due to the custom of women delivering their first baby at their parents' home. We included these covariates in the model to adjust for these differences but there is likely still residual confounding.

Conclusion

Using weights measured at or around the nadir – as typically occurs during community-based studies due to the logistics of quickly transporting data collection teams - over-estimates the prevalence of SGA and low birthweight. We found a lower prevalence when using birthweight imputed to the time of delivery compared to weight taken within 72 hours post-delivery in a sample of deliveries in rural Nepal – an area where less than half of women deliver at facilities, high levels of exclusive breastfeeding and only half of babies are born with an appropriate weight for gestational age. Other studies using birthweight measurements taken from infants born at home should consider a similar imputation model to generate more accurate SGA/low birth weight status at the population level.

Overall, more research on improving estimates of SGA is needed. This model may be generalizable to other South Asian LMIC countries but may not be in LMICs with lower SGA prevalence, such as Sub-Saharan Africa. Cohort studies with birthweight measurements should consider measuring longitudinal, early neonatal weight change since data in more diverse settings is needed, especially if a similar birthweight imputation is planned. An imputation model may also be clinically useful to better discriminate smaller babies, improving specificity of care. Finally, we note this model is restricted to livebirths since neither study measured weights on stillbirths. It would be culturally inappropriate to ask families to retain the fetal remained until study staff could arrive for weight measurement. However, IUGR may be an important cause of fetal death in this area and excluding stillbirths will likely under-estimate the prevalence of SGA.

For peer review only - http://bmjopen.bmj.com/site/about/guidelines.xhtml

Our study could not address this but including stillbirths in global estimates is critical to understanding the burden of IUGR and further work is needed to determine whether stillbirth weights may be imputed.

For peer review only

For peer review only - http://bmjopen.bmj.com/site/about/guidelines.xhtml

Contributors' Statement:

Prof. Katz conceptualized and designed the study, designed the data collection protocols, developed the analytic plan and statistical models, and reviewed and revised the manuscript.

Prof. Mullany conceptualized and designed the study, designed the data collection protocols, revised data collection tools and supervised data collection, developed the analytic plan and statistical models, and reviewed and revised the manuscript.

Prof. Tielsch conceptualized and designed the study, designed the data collection protocols, and reviewed and revised the manuscript.

Dr. Khatry revised data collection tools and supervised data collection and reviewed and revised the manuscript.

Prof. Zeger developed the analytic plan and statistical models and reviewed and revised the manuscript.

Dr. Mohan, Ms. Subedi contributed significantly to the analysis and critically reviewed and revised the manuscript for important intellectual content.

Dr. Hazel developed the analytic plan and statistical models, carried out the analysis and drafted the initial manuscript.

All authors reviewed and approved the final manuscript as submitted and agree to be accountable for all aspects of the work in ensuring that questions related to the accuracy or integrity of any part of the work are appropriately investigated and resolved.

Competing Interest: The authors have indicated they have no conflicts of interest, financial relationships, or competing interests relevant to this article to disclose.

For peer review only - http://bmjopen.bmj.com/site/about/guidelines.xhtml

334 **Funding:** This work was supported by the National Institute for Child Health and Human Development (R01HD092411 and R01HD060712), the Bill & Melinda Gates Foundation (OPP1084399), National Institutes of health (810-2054), and Cooperative Agreements HRN-A-00-97-00015-00 and GHS-A-00-03-000019-00 between Johns Hopkins University and the Office of Health and Nutrition, US Agency for International Development. Under the grant conditions of the Foundation, a Creative Commons Attribution 4.0 Generic License has already been assigned to the Author Accepted Manuscript version that might arise from this submission. The funder had no role in the study design, collection or analysis of data, writing of the report or the decision to submit the article for publication.

343 **Data sharing statement:** No additional data available

For peer review only

References:

1. Lee ACC, Katz J, Blencowe H, et al. National and regional estimates of term and preterm babies born small for gestational age in 138 low-income and middle-income countries in 2010. *Lancet Glob Health.* 2013;1(1).

2. Katz J, Lee AC, Kozuki N, et al. Mortality risk in preterm and small-for-gestational-age infants in low-income and middle-income countries: a pooled country analysis. *Lancet*. 2013;382(9890):417-425.

3. Hughes MM, Black RE, Katz J. 2500-g Low Birth Weight Cutoff: History and Implications for Future Research and Policy. *Matern Child Health J.* 2017;21(2):283-289.

4. DiTomasso D, Cloud M. Systematic Review of Expected Weight Changes After Birth for Full-Term, Breastfed Newborns. *J Obstet Gynecol Neonatal Nurs.* 2019;48(6):593-603.

5. Chauhan SP, Rice MM, Grobman WA, et al. Neonatal Morbidity of Small- and Large-for-Gestational-Age Neonates Born at Term in Uncomplicated Pregnancies. *Obstet Gynecol.* 2017;130(3):511-519.

6. Mendez-Figueroa H, Truong VTT, Pedroza C, Chauhan SP. Large for Gestational Age Infants and Adverse Outcomes among Uncomplicated Pregnancies at Term. *Am J Perinatol.* 2017;34(7):655-662.

7. Lee AC, Kozuki N, Cousens S, et al. Estimates of burden and consequences of infants born small for gestational age in low and middle income countries with INTERGROWTH-21st standard: analysis of CHERG datasets. *BMJ*. 2017;358:j3677.

8. Mullany LC, Darmstadt GL, Khatry SK, et al. Topical applications of chlorhexidine to the umbilical cord for prevention of omphalitis and neonatal mortality in southern Nepal: a community-based, cluster-randomised trial. *The Lancet*. 2006;367(9514):910-918.

9. Tielsch JM, Darmstadt GL, Mullany LC, et al. Impact of Newborn Skin-Cleansing With Chlorhexidine on Neonatal Mortality in Southern Nepal: A Community-Based, Cluster-Randomized Trial. *Pediatrics*. 2007;119(2):e330-e340.

10. Ministry of Health, Nepal, New ERA, and ICF. Nepal Demographic and Health Survey 2016. 2017. Accessed January 13, 2020. https://dhsprogram.com/pubs/pdf/FR336/FR336.pdf

11. Papageorghiou AT, Kennedy SH, Salomon LJ, et al. The INTERGROWTH-21st fetal growth standards: toward the global integration of pregnancy and pediatric care. *Am J Obstet Gynecol*. 2018;218(2S):S630-S640.

12. StataCorp. Stata Statistical Software: Release 16.; 2019.

13. Fonseca MJ, Severo M, Santos AC. A new approach to estimating weight change and its reference intervals during the first 96 hours of life. *Acta Paediatr*. 2015;104(10):1028-1034.

14. Modi N, Bétrémieux P, Midgley J, Hartnoll G. Postnatal weight loss and contraction of the extracellular compartment is triggered by atrial natriuretic peptide. *Early Hum Dev*. 2000;59(3):201-208.

15. Singhi S, Sood V, Bhakoo ON, Ganguly NK, Kaur A. Composition of postnatal weight loss & subsequent weight gain in preterm infants. *Indian J Med Res*. 1995;101:157-162.

For peer review only - http://bmjopen.bmj.com/site/about/guidelines.xhtml

16. Bauer K, Versmold H. Postnatal weight loss in preterm neonates less than 1,500 g is due to isotonic dehydration of the extracellular volume. *Acta Paediatr Scand Suppl*. 1989;360:37-42.

17. vd Wagen A, Okken A, Zweens J, Zijlstra WG. Composition of postnatal weight loss and subsequent weight gain in small for dates newborn infants. *Acta Paediatr Scand*. 1985;74(1):57-61.

18. Bauer K, Cowett RM, Howard GM, vanEpp J, Oh W. Effect of intrauterine growth retardation on postnatal weight change in preterm infants. *J Pediatr*. 1993;123(2):301-306.

19. Pandey S. Women's knowledge about the conditional cash incentive program and its association with institutional delivery in Nepal. *PLOS ONE*. 2018;13(6).

For peer review only

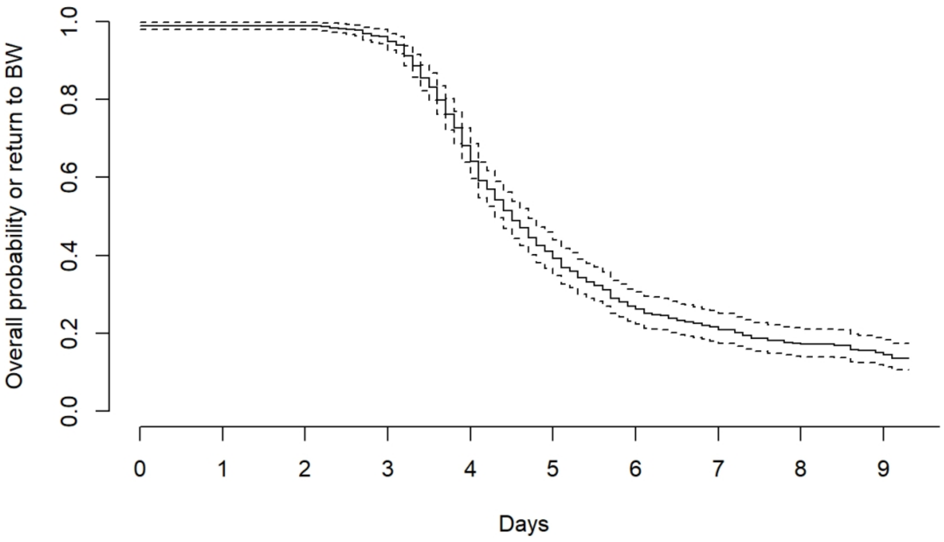

Figure 1: Probability of return-to-birthweight and 95% confidence intervals, chlorhexidine trial. Note: Babies that did not return to their birthweight in the 10-day period were censored.

467x289mm (76 x 76 DPI)

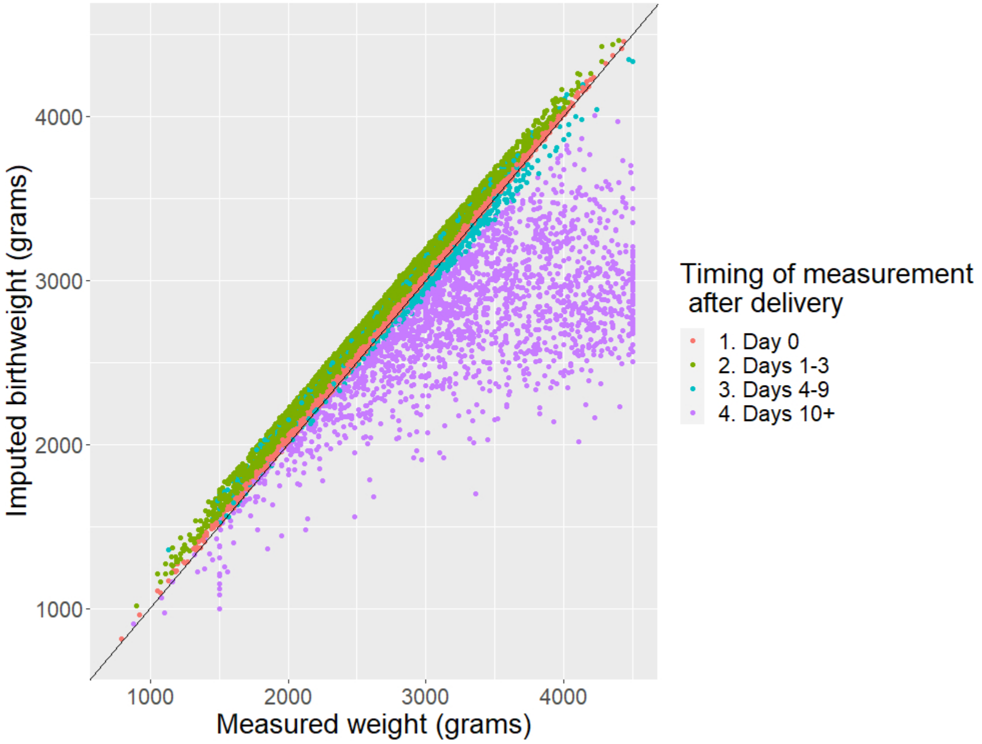

Figure 2: Comparison of measured weight and imputed weight in grams by the timing of the weight measurement in days after delivery, NOMS dataset.

651x488mm (39 x 39 DPI)

Annexes:

Table A1: Regression outputs for the training model, chlorhexidine trial (n= 4148 weight measurements on 456 infants)

	Weight, grams (standard error)
Parity<br>(reference=previous birth)	-163.7 (49.6)
Sex<br>(reference=female)	50.6 (37.7)
Gestational age in weeks, centered at 40 weeks	52.7 (7.9)
Maternal education in years	-0.5 (3.3)
Maternal age in years	10.7 (6.0)
Intercept	2745.1 (90.0)

Table A2: Proportion of infants with weight missing or weight measured greater than 72 hours post-delivery stratified by preterm birth, survival status, and maternal parity and education, NOMS data

	**Type of missingness % (95% CI)**	
**Proportion missing with:**	**Weight missing**	**Weight measured greater than 72 hours after delivery**
Preterm birth (<37 weeks)	16.3<br>(15.3 – 17.4)	13.6<br>(12.6 – 14.7)
Non-preterm birth (>=37 weeks)	11.7<br>(11.3 – 12.1)	13.5<br>(13.1 – 14.0)
Infant alive at end of study	10.6<br>(10.3 – 11.0)	13.6<br>(13.2 – 14.1)
Infant deceased at end of study	61.7<br>(58.7 – 64.6)	4.8<br>(3.1 – 7.4)
No previous births	15.7<br>(15.0 – 16.4)	17.0<br>(16.2 – 17.8)
Any previous births	10.9<br>(10.5 – 11.3)	12.0<br>(11.5 – 12.5)
No education	11.2<br>(10.8 – 11.7)	11.6<br>(11.2 – 12.1)
Any education	14.9<br>(14.2 – 15.6)	17.6<br>16.8 – 18.4)

CI: confidence intervals

For peer review only

BMJ Open

An imputation model to recalibrate birthweights measured in the early neonatal period to time at delivery its impact on size-for-gestational age and low birthweight prevalence estimates: a secondary analysis of a pregnancy cohort in rural Nepal.

Journal:	*BMJ Open*
Manuscript ID	bmjopen-2021-060105.R1
Article Type:	Original research
Date Submitted by the Author:	09-May-2022

Complete List of Authors:	Hazel , Elizabeth; Johns Hopkins University Bloomberg School of Public Health,<br>Mullany, Luke C.; Johns Hopkins University Bloomberg School of Public Health, International Health<br>Zeger, Scott ; Johns Hopkins University Bloomberg School of Public Health<br>Mohan, Diwakar; Johns Hopkins University Bloomberg School of Public Health<br>Subedi, Seema; Johns Hopkins University Bloomberg School of Public Health, International Health<br>Tielsch, James; George Washington University School of Public Health and Health Services, Global Health<br>Khatry, Subarna; Nepal Nutr Intervent Project Sarlahi<br>Katz, Joanne; Johns Hopkins University Bloomberg School of Public Health, International Health

**Primary Subject Heading**:	Public health
Secondary Subject Heading:	Global health, Paediatrics, Research methods, Obstetrics and gynaecology
Keywords:	PUBLIC HEALTH, Maternal medicine < OBSTETRICS, NEONATOLOGY

SCHOLARONE™
Manuscripts

I, the Submitting Author has the right to grant and does grant on behalf of all authors of the Work (as defined in the below author licence), an exclusive licence and/or a non-exclusive licence for contributions from authors who are: i) UK Crown employees; ii) where BMJ has agreed a CC-BY licence shall apply, and/or iii) in accordance with the terms applicable for US Federal Government officers or employees acting as part of their official duties; on a worldwide, perpetual, irrevocable, royalty-free basis to BMJ Publishing Group Ltd ("BMJ") its licensees and where the relevant Journal is co-owned by BMJ to the co-owners of the Journal, to publish the Work in this journal and any other BMJ products and to exploit all rights, as set out in our licence.

The Submitting Author accepts and understands that any supply made under these terms is made by BMJ to the Submitting Author unless you are acting as an employee on behalf of your employer or a postgraduate student of an affiliated institution which is paying any applicable article publishing charge ("APC") for Open Access articles. Where the Submitting Author wishes to make the Work available on an Open Access basis (and intends to pay the relevant APC), the terms of reuse of such Open Access shall be governed by a Creative Commons licence – details of these licences and which Creative Commons licence will apply to this Work are set out in our licence referred to above.

Other than as permitted in any relevant BMJ Author's Self Archiving Policies, I confirm this Work has not been accepted for publication elsewhere, is not being considered for publication elsewhere and does not duplicate material already published. I confirm all authors consent to publication of this Work and authorise the granting of this licence.

For peer review only

An imputation model to recalibrate birthweights measured in the early neonatal period to time at delivery its impact on size-for-gestational age and low birthweight prevalence estimates: a secondary analysis of a pregnancy cohort in rural Nepal.

Elizabeth Hazel[a], PhD, Luke C. Mullany[a], PhD, Scott L. Zeger[a], PhD, Diwakar Mohan[a], DrPH MD,Seema Subedi[a], MPH, James M. Tielsch[b], PhD MHSc, Subarna K. Khatry[a,c], MBBS, Joanne Katz[a], ScD.

Affiliations: [a]Johns Hopkins Bloomberg School of Public Health, Baltimore, Maryland, USA; [b]George Washington University Milken Institute School of Public Health, Washington DC, USA; and [c]Nepal Nutrition Intervention Project-Sarlahi (NNIPS), Sarlahi, Nepal.

Address correspondence to: Elizabeth Hazel, Department of International Health, Johns Hopkins Bloomberg School of Public Health, 615 N. Wolfe St. Room W5504**,** Baltimore, MD 21205, USA, ehazel1@jhu.edu, +14106148821

Acknowledgements:

We would like to acknowledge Prof. Anne CC Lee at Harvard Medical School and Prof. Robert Black at Johns Hopkins Bloomberg School of Public Health for their technical contributions, and the study staff and participants at the Nepal Nutritional Intervention Project-Sarlahi site.

For peer review only - http://bmjopen.bmj.com/site/about/guidelines.xhtml

Abstract

Objectives: In low-income countries, birthweights for home deliveries are often measured at the nadir when babies may lose up of 10% of their birthweight, biasing prevalence estimates of small-for-gestational age (SGA) and low birthweight (LBW). We aimed to develop a model that predicts the "true" birthweight at time of delivery.

Design: Application of a model that recalibrates weights measured in the early neonatal period to time=0 at delivery and uses those recalibrated birthweights to the multiply impute for babies with missing birthweight.

Setting: This is a secondary analysis using pregnancy cohort data from two studies in Sarlahi district, Nepal.

Participants: The participants are 457 neonates with longitudinal weight data from a subsample of a larger clinical trial on chlorhexidine (CHX) neonatal skin cleansing and 30,680 neonates with weights measured throughout the early neonatal period (Nepal Oil Massage Study (NOMS)).

Primary and secondary outcome measures: We developed an empirical Bayes model of early neonatal weight change using CHX trial longitudinal data and applied it to the NOMS dataset to recalibrate and impute birthweight at delivery. The primary outcomes are the differences in SGA and LBW prevalence using imputed and measured birthweights.

Results: When using the imputed birthweights, the proportion of SGA is reduced from 49% (95% CI: 48-49%) to 44% (95% CI: 43-44%), a relative reduction of 10%. Low birthweight is reduced by 11%, from 30% (95% CI: 30-31%) to 27% (95% CI: 26-27%). The proportion of babies born large-for-gestational age increased from 4% (95% CI: 3.6-4.1) to 5% (95% CI: 4.5-5.0) or a relative increase of 24%.

For peer review only - http://bmjopen.bmj.com/site/about/guidelines.xhtml

Conclusions: Using weights measured around the nadir over-estimates the prevalence of SGA and LBW. Studies in low-income settings with high levels of home births should consider a similar recalibration and imputation model to generate more accurate population estimates of vulnerable newborns.

Article Summary

Strengths and limitations of this study

- One strength of this study is that we developed our model of early neonatal growth using longitudinal data of daily weight change.
- Another strength is that the data used to develop the model was sampled from a similar population of infants in rural Nepal as was the study used to apply the model; although there may be differences due to secular trends, the median birthweights and neonatal mortality rates of the two samples are similar.
- Another strength is that both studies included high quality gestational age and weight measurements taken on a population-representation sample with relatively low levels of missing data.
- One limitation of the study is that the birthweights are modelled and not empirical data.
- Another limitation is that the model may not be generalizable to low-income settings outside of South Asia where prevalence of intrauterine growth restriction is lower.

For peer review only - http://bmjopen.bmj.com/site/about/guidelines.xhtml

Introduction

In 2010, 32.4 million babies in low-and-middle income countries (LMICs) were estimated to be small-for-gestational age (SGA), over a quarter of all live births and these babies had an 83% higher risk of dying the first month of life.[1,2] The South Asia region has the highest prevalence of SGA in the world; a third of babies are born small (34%) compared to the global rate (19%).[3] SGA is defined as a birthweight-for-gestational age less than the 10th percentile of a standard infant reference population and severe SGA is less that the 3rd percentile. A low weight at birth (defined as babies born less than 2500g regardless of gestational age) may result from a preterm birth (births less than 37 completed weeks gestational age), intra-uterine growth restriction (IUGR), or a combination of the two. The etiology of babies born with IUGR is likely distinct from those born preterm, and perhaps even different from babies born early with IUGR. To better understand the epidemiology of low birthweight babies, practitioners and researchers have moved to monitoring birthweight by gestational age, although low birthweight prevalence is still appropriate to use in settings where gestational age measured by ultra-sound in the first trimester (the gold standard) is rare.[4] Some babies will be on the smaller end of the curve naturally, but in LMICs where SGA estimates are high, it can be used as a proxy for IUGR prevalence. Large-for-gestational age (LGA) or babies with a birthweight >90th centile have associated health risks. [5,6] SGA, LGA and low birthweight prevalence in LMICs are essential indicators of population health.

Along with gestational age, an accurate birthweight measured close to the time of birth is critical for estimating SGA. This is because neonatal weight decreases for the first 2-3 days after birth (known as the *nadir* or period of minimum neonatal weight), before starting to increase. A systematic review of breastfed, healthy, singleton babies from high income countries found the nadir of mean weight loss of 7-8% from birthweight at day three. Many neonates in these studies

For peer review only - http://bmjopen.bmj.com/site/about/guidelines.xhtml

had weight loss greater than 7% and up to 10% from birthweight for exclusively breastfed babies.[7]

Birthweight is typically measured shortly after delivery for babies born at health facilities. For babies born outside of facilities in LMICs, it may take 24 to 72 hours before a health worker can measure weight, often at the nadir of the infant's weight loss. Weights of babies taken during the nadir may overestimate SGA and low birthweight prevalence and underestimate LGA.

In this study, we aimed to generate estimates of SGA, LGA, and low birthweight using an imputation model that extrapolates infant weights measured post-delivery (around the nadir) to a recalibrated weight at the time of birth and then uses those recalibrated birthweight to impute for babies missing a weight measurement.

Methods

Data sources

This was a secondary data analysis of two pregnancy cohort datasets from rural Nepal (table 1). The training dataset was derived from a cluster-randomized community-based trial examining the effect of chlorhexidine full body wipe at delivery and chlorhexidine umbilical stump application on neonatal omphalitis and neonatal mortality (CHX). [8,9] From 2002 to 2005, pregnant women were recruited approximately in the sixth month of pregnancy and infants were followed-up on 1, 3, 7, 10, 14, 21 and 28 of days of age. The dataset where we imputed weight at delivery was from another cluster-randomized community-based trial called the Nepal Oil Massage Study (NOMS), (clinicaltrials.gov #NCT01177111). From 2010-2017, pregnant women were enrolled through community surveillance, and their newborns were randomized to receive postnatal massage using either sunflower or mustard seed oil to determine the impact of oil type on neonatal morbidity and

For peer review only - http://bmjopen.bmj.com/site/about/guidelines.xhtml

108 mortality. Both studies took place in rural Sarlahi district in southern Nepal. In this area, less than half of the women delivery in health facilities (40%), c-sections are very low (<1%) and generally 90% of infants are breastfed within the first day of delivery. [10]

111 **Table 1: Description of data sets used for analysis.**

	**Training data**	**Study data**
Name	Chlorhexidine trial (CHX)	Oil massage trial (NOMS)
Setting	Sarlahi district, Nepal	Sarlahi district, Nepal
Years of data collection	2002-05	2010-17
Number of livebirths included in the analysis	457	30,680
Male gender (%)	57.6<br>95% CI: 53.0 – 62.0	51.9<br>95% CI: 51.3 – 52.4
Median gestational age at delivery (weeks)	39.7<br>IQR: 37.8 – 41.2	39.4<br>IQR: 38.0 – 40.9
Median birthweight (non-imputed, grams)	2706<br>IQR: 2480 – 2986	2740<br>IQR: 2450 - 3040
Time of first weight measurement after birth (hours)		
Median	4.1<br>IQR: 3.1 – 5.2	15.2<br>IQR: 7.9 – 25.3
Mean	4.0<br>SD: 1.28	89.1<br>SD: 275.42
Pre-term, <37 weeks gestational age (%)	14.9<br>95% CI 11.9 – 18.5	15.7<br>95% CI: 15.3 – 16.1
Health facility delivery (%)	3.7<br>95% CI 2.3-5.9	58.1<br>95% CI 57.6-58.7
Gestational age ascertainment	Last menstrual period	Last menstrual period
Instrument used to measure infant weight	Seca Digital Scale Model 727 and Tanita digital infant weight scale	Tanita digital infant weight scale

112

113 In the CHX trial, a subset of infants (n=457) with first weight measured in the first six hours after delivery were purposively sampled for the longitudinal sub-study, and then followed for daily weight measurements through the first 10 days of life. Gestational age was calculated using date of last menstrual period (LMP). Multiples and babies that did not survive the early neonatal period were not included in the CHX subsample of longitudinal data. We selected this data to model

For peer review only - http://bmjopen.bmj.com/site/about/guidelines.xhtml

early neonatal weight change since it drew from the same population but earlier timepoint as the NOMS study.

For the NOMS study, women in the study area were visited approximately every five weeks and asked about the timing of their LMP. Pregnant women were enrolled throughout the pregnancy (median gestational age at enrollment was 14.1 weeks) and some women contributed multiple pregnancies to the study data. Birthweight was collected at the first postnatal visit on all surviving liveborn infants. Information on maternal age, parity, education, household wealth and infant sex and survival status were collected through interviews with the mother. In both studies, birthweight was measured by trained study staff using digital scales.

Descriptive analysis

For the CHX longitudinal data, we calculated the median and interquartile range for nadir weight, time in days to reach the nadir, and time in days to return to the birthweight in the 10-day period for the CHX study. To make these estimates, we developed a function that extends the predicted weights from time of delivery to a maximum observed time in units of days (within the 10-day period). The function then finds the time in days when the predicted weight achieves its minimum and when the weight returns closest to the birth weight. We plotted a survival curve for probability of return to birthweight within the 10 days. Babies that did not return to their birthweight in the 10-day period were censored.

For the NOMS data, we described the sample and analyzed the pattern of missing weights and weights taken greater than 72 hours after delivery to determine if babies with missing and non-missing weights differed by preterm status (<37 weeks), survival status through the neonatal period, and mother’s parity and educational level.

Predicting birthweight

For peer review only

We imputed weight at birth in the NOMS dataset by estimating, then recalibrating from the conditional distribution of each child's birthweight (weight at t=0) given a single measurement at a known later time t>0 (case 1) or imputing given missing weight (case 2).

In both cases, the strategy had three steps: (1) use the longitudinal CHX study to estimate the dependence of birthweight on later weights and assume this relationship holds approximately for the NOMS data as well; (2) use the NOMS data to model the dependence of population average weight on age at measurement and other key predictors; (3) integrate (1) and (2) to obtain the optimal linear predictor of birthweights in NOMS given the single weight measurement on each child at age t.

$$Yij = \beta_0 + \beta_1 X_{1ij} + \ldots \beta_p X_{pij} + u_{0j} + \ldots u_{ij} X_{1ij} + e_{ij}$$

For the first step, the CHX data was used to fit a linear mixed effect model with the repeated weights as the outcomes (Y is the daily weight for j^{th} of n_i for each baby). The fixed effects included parity, maternal education/age, infant sex, and gestational age at birth in weeks with a spline at 40 weeks. The random effects within baby include intercept and linear age.

This model enabled us to estimate the conditional distribution of the unknown birthweight given one or more weights at later times, controlling for fixed effects. The mean of this distribution is the empirical Bayes estimate of birthweight. We applied the variance parameters from the training model to NOMS data to generate the distribution of the imputed birthweights for NOMS (Box 1).

For the second step, using NOMS data, we regressed the observed weight (w_t) at age t in hours on: a smooth function of maternal age and education in years, the neonate's survival status by day of age, infant sex, an indicator of preterm birth, and whether the birth was a multiple or singleton, and whether it was the mother's first birth (parity) using a linear mixed effect model (Y the

For peer review only - http://bmjopen.bmj.com/site/about/guidelines.xhtml

estimated weight for a ith woman's j[th] pregnancy that resulted in a livebirth with a measured weight). Neonatal survival status was defined as a liveborn death in the first 28 days of life with the reference being children who survived the neonatal period (28 days after birth) or alive at the time they were lost-to-follow-up. We used this model to obtain the estimated weight (w_{it}^{NOMS}) for

Box 1: Formulas for calculating the empirical Bayes estimate of weight and its variance, t=0

w_{it}= measured weight for child *i* at time *t>0*, NOMS

w_{it}^{NOMS} = predicted weight at actual measurement time (t>0), NOMS

w_{i0}^{NOMS} = predicted weight, t=0 from the linear mixed effect model, NOMS

G = 2x2 variance-covariance matrix of random effects in the linear mixed effects model, CHX

σ^2= residual variance from the linear mixed effects model, CHX

$Z_i = n_i \times 2$ random effects design matrix where n_i is the number of ages from which are being used to predict the birthweight, here n_i=1 and $Z_i=(1, age_i)$, NOMS

$v_i = Z_i G t(Z_i)$ = variance of the linear combination of the random effects that contribute to the birthweight prediction, NOMS, CHX

w_{i0}^{NOMS} = empirical Bayes estimate of birthweight = $w_{i0}^{NOMS} + v_i^2/(v_i + \sigma^2)(w_{it} - w_{it}^{NOMS})$

$Var(w_{i0}^{NOMS}) = (G_{11} + \sigma^2) - v_i^2/(v_i + \sigma^2)$

infant *i* at their measurement time *t* and their predicted birth weight (w_{i0}^{NOMS}) at *t*=0 (Box 1).

In the final step (3), we applied the relationship between birthweight and subsequent weights derived in step (1) to the NOMS observed and predicted weights to obtain an improved prediction (recalibration) of the NOMS infant's birthweights (Box 1). We generated m=5 random Gaussian variates of the imputed birthweight.

We used these recalibrated weights to impute for infants with missing weight data (case 2). We fit a random forest model to the recalibrated birthweight dataset with fixed effects of baby's gender, gestational age at delivery, multiple/singleton, neonatal survival status, and maternal parity, education, and age. Using this model, we then imputed birthweight for babies with no weights measured and randomly generated a m=5 dataset for imputation. Infants with missing

For peer review only - http://bmjopen.bmj.com/site/about/guidelines.xhtml

parity, survival status, maternal age/education, gestational age, singleton/multiples, or sex data were excluded from the imputation. All modelling for case 1 and case 2 were performed in R. [11]

Comparing size-for-gestational age and low birth weight using raw and imputed weights

We generated appropriate for gestational age (AGA) (10th – 90th centiles), SGA (10th centile), and LGA (90th centile) using Intergrowth-21 standards extrapolated up to 22+0 to 44+6 weeks GA[1] (original model was 24+0 to 42+6 weeks). [12] We generated imputed estimates of AGA, SGA, LGA and low birthweight (< 2500g) and very low birthweight (<1500g) prevalence with multiple imputation (m=5) in Stata 16.1. [13] The main outcome was the prevalence estimate comparison using the imputed birthweight versus measured weight within 72 hours. As a sensitivity analysis, we compared the proportion of term and preterm babies, female and male gender babies, and the neonatal mortality rate using the dataset with the raw birthweight versus the imputed dataset so see whether babies are being included or excluded with the imputation.

Ethical considerations

This is a secondary data analysis study of two trials for which institutional review board approvals were obtained from the Johns Hopkins Bloomberg School of Public Health (FWA00000287) and Nepal Health Research Council (FWA 00000957). All participants gave their informed consent to take part in both studies. This secondary data analysis was considered exempt by the Johns Hopkins Bloomberg School of Public Health institutional review board and conforms to the principles embodied in the Declaration of Helsinki.

Patient and Public Involvement

For peer review only

[1] Personal Communication, Eric Ohuma, London School of Hygiene and Tropical Medicine, September 2021

For peer review only - http://bmjopen.bmj.com/site/about/guidelines.xhtml

The patients and public were not involved in the design, conduct, reporting or dissemination of this research. Accurately measuring the health and nutritional status of infants is important to clinicians, researchers and families in Nepal, however, we did not seek the public's priorities, experience or preferences when designing this research question. We did not contact the original participants in the two studies used in this secondary data analysis.

Results

The median birthweight in the CHX trial (training data) was 2718 (IQR 2482 – 2981) (table 2). The median time of nadir was 2.1 days and the median weight at the nadir was 2607 (2773 – 2854); a relative weight loss of 2% or 55 grams. Median return to birthweight was 4.4 days (fig. 1). Eighty-four infants did not regain their birthweight with the 10 days.

Table 2: Neonatal growth during the first 10 days of life, chlorhexidine trial (n= 4148 weight measurements on 456 infants)

	Median (IQR)
Birthweight (grams)	2718 (2482 – 2982)
Nadir weight (grams)	2607 (2373 – 2854)
Time to reach nadir (days)	2.1 (1.9 – 2.3)
Time to return to birthweight (days)	4.4 (3.7 – 6.0)
Number of babies that did not return to birthweight within the 10 days	84

IQR: Interquartile range

From the model outputs, we found babies born to mothers with no previous births were estimated to have a lower than average birthweight and baby girls born at a greater gestational age born to older mothers were estimated to have higher birthweights on average (Annex table A1). There was minimal impact of maternal education on birthweight adjusted by other covariates. We

For peer review only - http://bmjopen.bmj.com/site/about/guidelines.xhtml

compared this model to others excluding different maternal level factors and full model has the best fit with small differences in the Akaike's *An Information Criteria* (AIC) (Annex table A2).

There were 31,116 livebirths from 22,495 women in the NOMS dataset (table 3). We imputed a birthweight on 99% of the livebirths (n=30,996) with complete covariate data and gestational age data. Of those livebirths, 3,863 were missing a weight measurement and we were able to impute a birthweight for 98% (n=3,769). The pattern of missing birthweight was not random (Annex table A3). Infants without a measured weight were more likely to be born to nulliparous mothers, with more education. Infants who were preterm were more likely to have a missing birthweight (16% preterm compared to 12% full term) and over half of deceased infants did not have a weight measure (62%) compared to a tenth of infants who survived during the neonatal period (11%). Those infants with a weight measured after 72 hours showed a similar pattern.

Table 3: Description of covariate data for all live births, NOMS study

		**All live births (n=31,116) % (n)**
Maternal age at LMP (years)		
	<18	15.5 (4,823)
	18-35	82.3 (25,622)
	>35	2.2 (671)
	Missing	0
Maternal education		
	No school	67.0 (20,855)
	1-5 years	8.5 (2,638)
	>5 years	24.4 (7,595)
	Missing	0.1 (28)
Parity (live or stillbirth)		
	No previous births	31.5 (9,794)
	1	27.4 (8,531)
	2	19.6 (6,108)
	3	10.8 (3,370)
	4+	10.1 (3,147)
	Missing	0.5 (166)
Gestational age		

For peer review only - http://bmjopen.bmj.com/site/about/guidelines.xhtml

Term (≥37 - <45 weeks)	84.3 (26,243)
Moderate-to-Late preterm (≥32-<37 weeks)	13.7 (4,262)
Early preterm (≥28-<32 weeks)	1.6 (501)
Extremely preterm (<28 weeks)	0.4 (110)
Missing	0
Infant sex	
Female	48.0 (14,925)
Male	51.7 (16,099)
Missing	0.3 (92)
Multiple births	
Singleton	98.4 (30,616)
Twin/ Triplets	1.6 (500)
Missing	0
Infant survival status	
Alive at 28 days	96.6 (30,041)
Lost-to-follow-up	3.5 (1,076)
Deceased by 28 days	3.3 (1,031)
Missing	(0.1) 44
Missing birth weight	12.4 (3,863)
Timing of birthweight measurement (Among non-missing birthweight, n=27,253)	
0-5 hours	17.5 (4,777)
6-23 hours	57.9 (15,773)
24-72 hours	11.1 (3,018)
>72 hours	13.5 (3,685)
Timing missing	0

For the NOMS imputation, at day 0 the imputed birthweights (mean 2768.6g, 95% CI: 2762.7-2774.5g) were on average 49.7g higher than the measured weights (mean 2718.9g, 95% CI: 2713.0 - 2724.7g) and on average 131g higher on days 1-3 ((fig. 2). During days 4-9 and 10+, when the baby begins to regain and surpass birthweight, the recalibration reduces the weights back down (on average a 22.8g reduction for measurements take on days 4-9 and an 856.5g average reduction for measurements take on day 10 or more after delivery (fig. 2). On average, babies that did not survive the neonatal period had an imputed weight 324g less than babies that did, and multiples had an imputed weight 659g less than singleton babies (Annex Table 1). There was a small amount

For peer review only

For peer review only - http://bmjopen.bmj.com/site/about/guidelines.xhtml

of heaping at 4500g for the measured weights; n=70 babies at exactly 4500g or a heaping index of 35% (+/- 250 grams) (fig. 2).

Using the imputed birthweights, we found differences in the SGA prevalence compared to weights taken within 72 hours after delivery (table 4). Using the imputed birthweight decreased the proportion of babies with low birthweight from 30% (95% CI: 30-31%) to 27% (95% CI: 26-27%). SGA prevalence using measured weights was 49% (95% CI: 48-49%) and 44% (95% CI: 43-44%) using imputed weights. The prevalence of LGA babies was similar in absolute terms (4% using the raw birthweights compared to 5% using the imputation). However, in relative terms, this is a 24% increase in the estimate of LGA from the measured to imputed. The proportion of low birthweight babies decreased from 30% (95% CI: 30-31%) to 27% (95% CI: 26-27%) and the proportion of very low birthweight babies (less than 1% of the sample) remained the same.

The sensitivity analysis showed no changes in baby gender or term or preterm delivery, except for very preterm. The proportion of babies born at 28-32 weeks increased significantly (95% CI not overlapping) using the imputed dataset. Also the neonatal mortality rate increased 109 percent from 16 deaths per 100 livebirths to 33 (Table 4). Among the babies with non-missing birthweights, the mean birthweight measured within 72 hours and the imputed birthweight was not statistically significantly different by survival status.

Table 4: **Comparison of low birthweight, small-for-gestational age, and large-for-gestational age prevalences.**

	Measured weight <72hr	Imputed birthweight	Relative change (%)	Absolute difference
Birthweight in grams; %, (95% CI)				
Low birthweight (<2500)	30.1 (29.5 – 30.7)	26.7 (26.0 – 27.4)	11.3	- 3.4
Very low birthweight (<1500g)	0.5 0.4 – 0.6	0.5 0.4 – 0.6	0	0
Size for gestational age in percentile; %, (95% CI)				

For peer review only - http://bmjopen.bmj.com/site/about/guidelines.xhtml

Appropriate for gestational age (90th - 10th centile)	47.7 (47.1 – 48.3)	51.6 (50.9 – 52.2)	8.2	+ 3.9
Small-for-gestational age (<10th centile)	48.5 (47.9 – 49.1)	43.7 (43.1 – 44.3)	10.0	- 4.8
Large-for-gestational age (>90th centile)	3.8 (3.6 – 4.1)	4.7 (4.5 – 5.0)	23.7	+1.0
Gestational age at delivery (weeks); % (95% CI)				
Term births (37 – 42)	74.3 73.7 - 74.9	73.3 72.8 - 73.8	1.3	-1.0
Moderate – to – late preterm (32-37)	13.4 13 - 13.8	13.7 13.3 - 14.1	2.2	+ 0.3
Very preterm (28 – 32)	1.3 1.2 - 1.4	1.6 1.5 - 1.8	23.7	+ 0.3
Extremely preterm (24 – 27)	0.3 0.2 - 0.3	0.4 0.3 - 0.4	33.3	+ 0.1
Post term (42-45)	10.8 10.4 - 11.2	11.0 10.6 - 11.3	1.9	+ 0.2
Female gender; %, (95% CI)	48.6 47.9 - 49.2	48.1 47.6 - 48.7	1.0	- 0.5
Male gender; %, (95% CI)	51.5 50.8 – 52.1	51.9 51.3 – 52.5	0.8	+ 0.4
Neonatal mortality rate; Deaths per 1000 livebirths (95% CI)	15.9 14.4 - 17.6	33.2 31.3 - 35.3	108.8	17.3
Birthweight in grams among babies with non-missing weight; mean (95% CI)				
Neonatal period survival	2713.2 2707.8 - 2718.7	2775.5 2770.0 - 2781.0	2.3	62.3
Neonatal death	2236.9 2171.8 - 2302.0	2290.0 2226.1 - 2353.9	2.4	53.1

CI: confidence intervals; pp: percentage points

Discussion

Birthweights measured during the nadir bias prevalence of SGA, LGA and low birthweight. The proportion of babies with SGA is reduced by a relative 10% and low birthweight by a relative 11% when including the birthweights imputed to time of delivery. Fonseca et al., modelled neonatal weight loss among full term, breastfed, singleton Portuguese infants with weight measured within 96 hours. [14] They fit several longitudinal models of weight change, but the goal was to examine the patterns and estimate a nadir, not to extrapolate the infant weights back to a "true" birthweight.

For peer review only - http://bmjopen.bmj.com/site/about/guidelines.xhtml

This model was done in a high income setting with low SGA prevalence and different patterns of facility delivery/c-section compared to many LMICs in South Asia.

In the first week after delivery, there is evidence that infants follow a broadly consistent pattern of weight change. Weight loss two to three days postnatally is primarily due to loss of water weight – specifically extracellular volume, likely the process of re-adapting the cardio-pulmonary system for life outside the womb. [15] Even babies born preterm lose weight primarily due to reduced extracellular volume. [16,17] However, IUGR babies may have a different pattern of weight change. One study of SGA/term and SGA/preterm babies found weight loss was due primarily to catabolism rather than loss of extracellular volume. [18] Another study comparing SGA to appropriate for gestational age, preterm babies found less postnatal diuresis in the SGA babies. [19] In settings with a high prevalence of SGA, population level estimates of postnatal growth in the first week are likely to be different compared to low SGA prevalence areas – due to different growth patterns in babies with IUGR. This model should be tested in other settings but has the potential to be used for correction of birthweight measured in the days post-delivery, particularly in South Asian LMICs where SGA prevalence is high compared to other regions.

The main strength of this study is that we had a very large dataset with a relatively small proportion of missing covariates. In both the CHX and NOMS studies, we have high quality gestational age at birth and infant weight data were taken on a population-representative sample of infants. The longitudinal training and analytical data were collected in the same geographic area from the same study site, and data collection was managed by the same research organization, the Nepal Nutritional Intervention Study Sarlahi (NNIPS). Another strength of this study is we were able to use daily, longitudinal weights to build the model of early neonatal growth.

For peer review only - http://bmjopen.bmj.com/site/about/guidelines.xhtml

One limitation is the differences between the training and analytical datasets. Difference in timing of data collection: CHX (2002 – 2005) versus NOMS (2010-2017). Babies born during the NOMS data collection may have a different weight change pattern compared to babies born in CHX trial due to secular trends in maternal health and nutritional status. The percent of women delivering at home has declined from CHX to NOMS, primarily due to the cash incentive program in Nepal that pays women to deliver in a facility. [20] However, the median birthweights (table 1) and the neonatal mortality rates are similar (30.06 neonatal deaths per 1000 livebirths in CHX and 31.18 per 1000 live births[2] in NOMS). [9]

All infants in the CHX subsample of longitudinal weights survived through the neonatal period and were singleton births. This is an important difference in the underlying population of the two samples (CHX versus NOMS) since multiples typically have lower birthweight and very SGA/preterm/low birth weight babies are more likely to die in the few days after delivery. The imputed and measured mean birthweights by survival status were similar, among the babies with non-missing birthweight, indicating that the imputation model performed well.

Another limitation is that the birthweights are not missing at random in NOMS. More than half the infants died in the NOMS study before their weight could be measured, and more preterm babies did not have a weight measurement (likely also due to early neonatal death). We found a higher proportion of very preterm babies in the sample with the imputed birthweights and a much higher neonatal mortality rate (table 4). We found infants of women with more education and wealth were missing birthweight, perhaps because they were more mobile and more likely to move out of the study area, or more likely to deliver at a facility and returned home after some days. Nulliparous women were also more likely to have no weights measured on their infants. This may

[2] Personal communication, Joanne Katz, Johns Hopkins Bloomberg School of Public Health November 2021

For peer review only - http://bmjopen.bmj.com/site/about/guidelines.xhtml

be due to the custom of women delivering their first baby at their parents' home. We included these covariates in the model to adjust for these differences but there is likely still residual confounding.

Conclusion

Using weights measured at or around the nadir – as typically occurs during community-based studies due to the logistics of quickly transporting data collection teams - over-estimates the prevalence of SGA and low birthweight. We found a lower prevalence when using birthweight imputed to the time of delivery compared to weight taken within 72 hours post-delivery in a sample of deliveries in rural Nepal – an area where less than half of women deliver at facilities, high levels of exclusive breastfeeding and only half of babies are born with an appropriate weight for gestational age. Other studies using birthweight measurements taken from infants born at home should consider a similar imputation model to generate more accurate SGA/low birth weight status at the population level.

Overall, more research on improving estimates of SGA is needed. This model may be generalizable to other South Asian LMIC countries but may not be in LMICs with lower SGA prevalence, such as Sub-Saharan Africa. Cohort studies with birthweight measurements should consider measuring longitudinal, early neonatal weight change since data in more diverse settings is needed, especially if a similar birthweight recalibration and imputation is planned. A recalibration model may also be clinically useful to better discriminate smaller babies, improving specificity of care. Finally, we note this model is restricted to livebirths since neither study measured weights on stillbirths. It would be culturally inappropriate to ask families to retain the fetal remains until study staff could arrive for weight measurement. However, IUGR may be an important cause of fetal death in this area and excluding stillbirths will likely under-estimate the

For peer review only - http://bmjopen.bmj.com/site/about/guidelines.xhtml

prevalence of SGA. Our study could not address this but including stillbirths in global estimates is critical to understanding the burden of IUGR and further work is needed to determine whether stillbirth weights may be imputed.

For peer review only

For peer review only - http://bmjopen.bmj.com/site/about/guidelines.xhtml

Contributors' Statement:

Prof. Katz conceptualized and designed the study, designed the data collection protocols, developed the analytic plan and statistical models, and reviewed and revised the manuscript.

Prof. Mullany conceptualized and designed the study, designed the data collection protocols, revised data collection tools and supervised data collection, developed the analytic plan and statistical models, and reviewed and revised the manuscript.

Prof. Tielsch conceptualized and designed the study, designed the data collection protocols, and reviewed and revised the manuscript.

Dr. Khatry revised data collection tools and supervised data collection and reviewed and revised the manuscript.

Prof. Zeger developed the analytic plan and statistical models and reviewed and revised the manuscript.

Dr. Mohan, Ms. Subedi contributed significantly to the analysis and critically reviewed and revised the manuscript for important intellectual content.

Dr. Hazel developed the analytic plan and statistical models, carried out the analysis and drafted the initial manuscript.

All authors reviewed and approved the final manuscript as submitted and agree to be accountable for all aspects of the work in ensuring that questions related to the accuracy or integrity of any part of the work are appropriately investigated and resolved.

Competing Interest: The authors have indicated they have no conflicts of interest, financial relationships, or competing interests relevant to this article to disclose.

For peer review only - http://bmjopen.bmj.com/site/about/guidelines.xhtml

Funding: This work was supported by the National Institute for Child Health and Human Development (R01HD092411 and R01HD060712), the Bill & Melinda Gates Foundation (OPP1084399), National Institutes of health (810-2054), and Cooperative Agreements HRN-A-00-97-00015-00 and GHS-A-00-03-000019-00 between Johns Hopkins University and the Office of Health and Nutrition, US Agency for International Development. Under the grant conditions of the Foundation, a Creative Commons Attribution 4.0 Generic License has already been assigned to the Author Accepted Manuscript version that might arise from this submission. The funder had no role in the study design, collection or analysis of data, writing of the report or the decision to submit the article for publication.

Data sharing statement: Data are available upon reasonable request.

For peer review only

References:

1. Lee ACC, Katz J, Blencowe H, et al. National and regional estimates of term and preterm babies born small for gestational age in 138 low-income and middle-income countries in 2010. *Lancet Glob Health.* 2013;1(1).

2. Katz J, Lee AC, Kozuki N, et al. Mortality risk in preterm and small-for-gestational-age infants in low-income and middle-income countries: a pooled country analysis. *Lancet.* 2013;382(9890):417-425.

3. Lee AC, Kozuki N, Cousens S, et al. Estimates of burden and consequences of infants born small for gestational age in low and middle income countries with INTERGROWTH-21st standard: analysis of CHERG datasets. *BMJ.* 2017;358:j3677.

4. Hughes MM, Black RE, Katz J. 2500-g Low Birth Weight Cutoff: History and Implications for Future Research and Policy. *Matern Child Health J.* 2017;21(2):283-289.

5. Chauhan SP, Rice MM, Grobman WA, et al. Neonatal Morbidity of Small- and Large-for-Gestational-Age Neonates Born at Term in Uncomplicated Pregnancies. *Obstet Gynecol.* 2017;130(3):511-519.

6. Mendez-Figueroa H, Truong VTT, Pedroza C, Chauhan SP. Large for Gestational Age Infants and Adverse Outcomes among Uncomplicated Pregnancies at Term. *Am J Perinatol.* 2017;34(7):655-662.

7. DiTomasso D, Cloud M. Systematic Review of Expected Weight Changes After Birth for Full-Term, Breastfed Newborns. *J Obstet Gynecol Neonatal Nurs.* 2019;48(6):593-603.

8. Mullany LC, Darmstadt GL, Khatry SK, et al. Topical applications of chlorhexidine to the umbilical cord for prevention of omphalitis and neonatal mortality in southern Nepal: a community-based, cluster-randomised trial. *The Lancet*. 2006;367(9514):910-918.

9. Tielsch JM, Darmstadt GL, Mullany LC, et al. Impact of Newborn Skin-Cleansing With Chlorhexidine on Neonatal Mortality in Southern Nepal: A Community-Based, Cluster-Randomized Trial. *Pediatrics*. 2007;119(2):e330-e340.

10. Ministry of Health, Nepal, New ERA, and ICF. Nepal Demographic and Health Survey 2016. 2017. Accessed January 13, 2020. https://dhsprogram.com/pubs/pdf/FR336/FR336.pdf.

11. R Core Team (2021). R: A language and environment for statistical computing. R Foundation for Statistical Computing, Vienna, Austria. URL https://www.R-project.org/

12. Papageorghiou AT, Kennedy SH, Salomon LJ, et al. The INTERGROWTH-21st fetal growth standards: toward the global integration of pregnancy and pediatric care. *Am J Obstet Gynecol*. 2018;218(2S):S630-S640.

13. StataCorp. Stata Statistical Software: Release 16.; 2019.

14. Fonseca MJ, Severo M, Santos AC. A new approach to estimating weight change and its reference intervals during the first 96 hours of life. *Acta Paediatr*. 2015;104(10):1028-1034.

15. Modi N, Bétrémieux P, Midgley J, Hartnoll G. Postnatal weight loss and contraction of the extracellular compartment is triggered by atrial natriuretic peptide. *Early Hum Dev*. 2000;59(3):201-208.

For peer review only - http://bmjopen.bmj.com/site/about/guidelines.xhtml

16. Singhi S, Sood V, Bhakoo ON, Ganguly NK, Kaur A. Composition of postnatal weight loss & subsequent weight gain in preterm infants. *Indian J Med Res*. 1995;101:157-162.

17. Bauer K, Versmold H. Postnatal weight loss in preterm neonates less than 1,500 g is due to isotonic dehydration of the extracellular volume. *Acta Paediatr Scand Suppl*. 1989;360:37-42.

18. vd Wagen A, Okken A, Zweens J, Zijlstra WG. Composition of postnatal weight loss and subsequent weight gain in small for dates newborn infants. *Acta Paediatr Scand*. 1985;74(1):57-61.

19. Bauer K, Cowett RM, Howard GM, vanEpp J, Oh W. Effect of intrauterine growth retardation on postnatal weight change in preterm infants. *J Pediatr*. 1993;123(2):301-306.

20. Pandey S. Women's knowledge about the conditional cash incentive program and its association with institutional delivery in Nepal. *PLOS ONE*. 2018;13(6).

For peer review only

For peer review only - http://bmjopen.bmj.com/site/about/guidelines.xhtml

Figure 1: Probability of return-to-birthweight and 95% confidence intervals, chlorhexidine trial.

Note: Babies that did not return to their birthweight in the 10-day period were censored.

Figure 2: Comparison of measured weight and imputed weight in grams by the timing of the weight measurement in days after delivery, NOMS dataset.

For peer review only

For peer review only - http://bmjopen.bmj.com/site/about/guidelines.xhtml

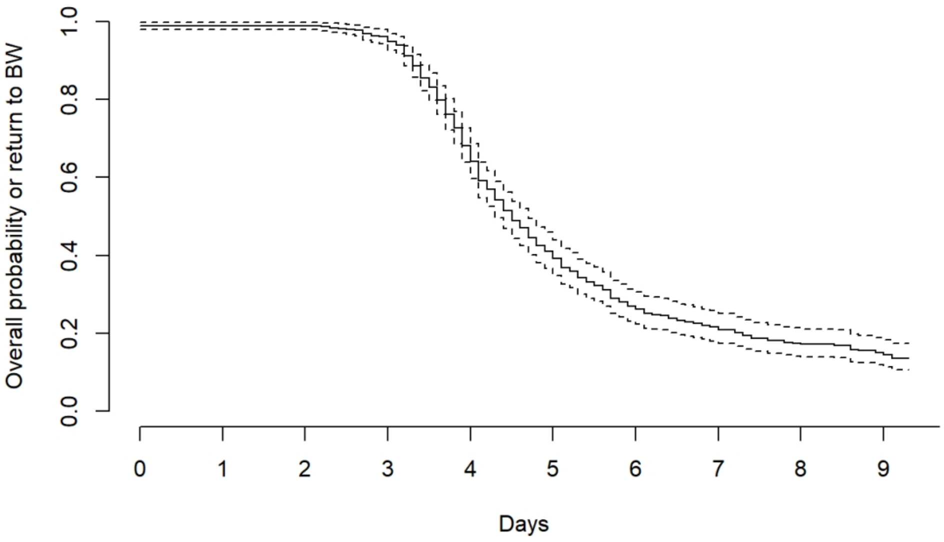

Figure 1: Probability of return-to-birthweight and 95% confidence intervals, chlorhexidine trial. Note: Babies that did not return to their birthweight in the 10-day period were censored.

467x289mm (76 x 76 DPI)

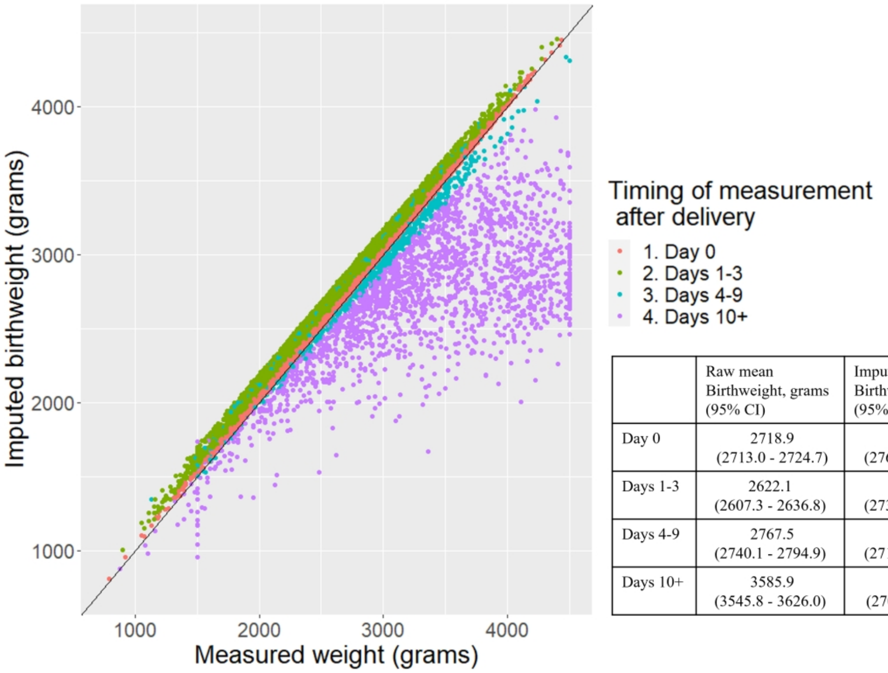

	Raw mean Birthweight, grams (95% CI)	Imputed mean Birthweight, grams (95% CI)
Day 0	2718.9 (2713.0 - 2724.7)	2768.6 (2762.7 - 2774.5)
Days 1-3	2622.1 (2607.3 - 2636.8)	2753.1 (2738.0 - 2768.2)
Days 4-9	2767.5 (2740.1 - 2794.9)	2744.7 (2716.2 - 2773.3)
Days 10+	3585.9 (3545.8 - 3626.0)	2729.4 (2700.8 - 2758.1)

Figure 2: Comparison of measured weight and imputed weight in grams by the timing of the weight measurement in days after delivery, NOMS dataset.

674x455mm (130 x 130 DPI)

Annexes:

Table A1: Regression outputs for the training model, chlorhexidine trial substudy (n= 4148 weight measurements on 456 infants) and the analytical model, NOMS trial (n=28016 infants, 27836 pregnancies and 21770 women)

	CHX Weight, grams (standard error)	*NOMS Weight, grams (standard error)*
Parity (reference=previous birth)	-163.7 (49.6)	-202.4<br>6.0
Sex (reference=female)	50.6 (37.7)	110.3<br>4.7
Gestational age in weeks, centered at 40 weeks	52.7 (7.9)	31.5<br>0.8
Neonatal death (reference = survives neonatal period or lost-to-follow-up)	N/A	-323.9<br>19.8
Maternal education in years	-0.5 (3.3)	13.8<br>0.7
Maternal age in years	10.7 (6.0)	5.4<br>0.6
Multiple gestation (twins or triplets) (reference = singleton)	N/A	-658.5<br>23.8
Intercept	2745.1 (90.0)	

N/A: there were no twins/triplets or neonatal deaths in the first 10 days in the CHX substudy.

Table A2: Akaike's *An Information Criteria* (AIC) for five models of predicted weight at delivery (training model), chlorhexidine trial (n= 4148 weight measurements on 456 infants)

Model	Covariates	Degrees freedom	AIC
Full model	Baby gender, Gestational age at delivery, Parity, Maternal age, and Maternal years of education,	13	48369.92
Model 1	Excluding Parity	12	48388.18
Model 2	Exclude Education	12	48376.52
Model 3	Exclude Education and Maternal age	11	48378.86
Model 4	Exclude: Education, Maternal age, and Parity	10	48396.61

AIC: Akaike's *An Information Criteria*

For peer review only - http://bmjopen.bmj.com/site/about/guidelines.xhtml

Table A3: Proportion of infants with weight missing or weight measured greater than 72 hours post-delivery stratified by preterm birth, survival status, and maternal parity and education, NOMS data

	**Type of missingness % (95% CI)**	
**Proportion missing with:**	**Weight missing**	**Weight measured greater than 72 hours after delivery**
Preterm birth (<37 weeks)	16.3 (15.3 – 17.4)	13.6 (12.6 – 14.7)
Non-preterm birth (>=37 weeks)	11.7 (11.3 – 12.1)	13.5 (13.1 – 14.0)
Infant alive at end of study	10.6 (10.3 – 11.0)	13.6 (13.2 – 14.1)
Infant deceased at end of study	61.7 (58.7 – 64.6)	4.8 (3.1 – 7.4)
No previous births	15.7 (15.0 – 16.4)	17.0 (16.2 – 17.8)
Any previous births	10.9 (10.5 – 11.3)	12.0 (11.5 – 12.5)
No education	11.2 (10.8 – 11.7)	11.6 (11.2 – 12.1)
Any education	14.9 (14.2 – 15.6)	17.6 16.8 – 18.4)

CI: confidence intervals

For peer review only

An imputation model to recalibrate birthweights measured in the early neonatal period to time at delivery its impact on size-for-gestational age and low birthweight prevalence estimates: a secondary analysis of a pregnancy cohort in rural Nepal.

Elizabeth Hazel[a], PhD, Luke C. Mullany[a], PhD, Scott L. Zeger[a], PhD, Diwakar Mohan[a], DrPH MD,Seema Subedi[a], MPH, James M. Tielsch[b], PhD MHSc, Subarna K. Khatry[a,c], MBBS, Joanne Katz[a], ScD.

STROBE Statement—Checklist of items that should be included in reports of ***cohort studies***

	Item No	Recommendation	Line number CLEAN version
**Title and abstract**	1	(*a*) Indicate the study's design with a commonly used term in the title or the abstract	1-3
		(*b*) Provide in the abstract an informative and balanced summary of what was done and what was found	22-52
**Introduction**			
Background/rationale	2	Explain the scientific background and rationale for the investigation being reported	69-95
Objectives	3	State specific objectives, including any prespecified hypotheses	96-99
**Methods**			
Study design	4	Present key elements of study design early in the paper	102
Setting	5	Describe the setting, locations, and relevant dates, including periods of recruitment, exposure, follow-up, and data collection	103 – 130, table 1
Participants	6	(*a*) Give the eligibility criteria, and the sources and methods of selection of participants. Describe methods of follow-up	103 – 130
		(*b*) For matched studies, give matching criteria and number of exposed and unexposed	Not applicable
Variables	7	Clearly define all outcomes, exposures, predictors, potential confounders, and effect modifiers. Give diagnostic criteria, if applicable	Primary and secondary outcomes: 184 – 192<br><br>Covariates: 155-170
Data sources/ measurement	8*	For each variable of interest, give sources of data and details of methods of assessment (measurement). Describe comparability of assessment methods if there is more than one group	103 – 130 Comparability table 1and Annex table 1
Bias	9	Describe any efforts to address potential sources of bias	Not applicable
Study size	10	Explain how the study size was arrived at	Available at citations of original studies
Quantitative variables	11	Explain how quantitative variables were handled in the analyses. If applicable, describe which groupings were chosen and why	155-170
Statistical methods	12	(*a*) Describe all statistical methods, including those used to control for confounding	144-182
		(*b*) Describe any methods used to examine subgroups and interactions	189-192

For peer review only - http://bmjopen.bmj.com/site/about/guidelines.xhtml

		(*c*) Explain how missing data were addressed	180-182
		(*d*) If applicable, explain how loss to follow-up was addressed	Not applicable
		(*e*) Describe any sensitivity analyses	189-192
**Results**			
Participants	13*	(a) Report numbers of individuals at each stage of study—eg numbers potentially eligible, examined for eligibility, confirmed eligible, included in the study, completing follow-up, and analysed	Available at citations of original studies
		(b) Give reasons for non-participation at each stage	Available at citations of original studies
		(c) Consider use of a flow diagram	Available at citations of original studies
Descriptive data	14*	(a) Give characteristics of study participants (eg demographic, clinical, social) and information on exposures and potential confounders	Available at citations of original studies
		(b) Indicate number of participants with missing data for each variable of interest	Available at citations of original studies
		(c) Summarise follow-up time (eg, average and total amount)	Available at citations of original studies
Outcome data	15*	Report numbers of outcome events or summary measures over time	Available at citations of original studies
Main results	16	(*a*) Give unadjusted estimates and, if applicable, confounder-adjusted estimates and their precision (eg, 95% confidence interval). Make clear which confounders were adjusted for and why they were included	232-250
		(*b*) Report category boundaries when continuous variables were categorized	Table 4
		(*c*) If relevant, consider translating estimates of relative risk into absolute risk for a meaningful time period	Not applicable
Other analyses	17	Report other analyses done—eg analyses of subgroups and interactions, and sensitivity analyses	251-256
**Discussion**			
Key results	18	Summarise key results with reference to study objectives	261-263
Limitations	19	Discuss limitations of the study, taking into account sources of potential bias or imprecision. Discuss both direction and magnitude of any potential bias	289-313
Interpretation	20	Give a cautious overall interpretation of results considering objectives, limitations, multiplicity of analyses, results from similar studies, and other relevant evidence	315-321
Generalisability	21	Discuss the generalisability (external validity) of the study results	324-328
**Other information**			
Funding	22	Give the source of funding and the role of the funders for the present study and, if applicable, for the original study on which the present article is based	328-366

*Give information separately for exposed and unexposed groups.

For peer review only

For peer review only - http://bmjopen.bmj.com/site/about/guidelines.xhtml

Note: An Explanation and Elaboration article discusses each checklist item and gives methodological background and published examples of transparent reporting. The STROBE checklist is best used in conjunction with this article (freely available on the Web sites of PLoS Medicine at http://www.plosmedicine.org/, Annals of Internal Medicine at http://www.annals.org/, and Epidemiology at http://www.epidem.com/). Information on the STROBE Initiative is available at http://www.strobe-statement.org.

For peer review only

BMJ Open

Development of an imputation model to recalibrate birthweights measured in the early neonatal period to time at delivery and assessment of its impact on size-for-gestational age and low birthweight prevalence estimates: a secondary analysis of a pregnancy cohort in rural Nepal

Journal:	*BMJ Open*
Manuscript ID	bmjopen-2021-060105.R2
Article Type:	Original research
Date Submitted by the Author:	10-Jun-2022

Complete List of Authors:	Hazel , Elizabeth; Johns Hopkins University Bloomberg School of Public Health,<br>Mullany, Luke C.; Johns Hopkins University Bloomberg School of Public Health, International Health<br>Zeger, Scott ; Johns Hopkins University Bloomberg School of Public Health<br>Mohan, Diwakar; Johns Hopkins University Bloomberg School of Public Health<br>Subedi, Seema; Johns Hopkins University Bloomberg School of Public Health, International Health<br>Tielsch, James; George Washington University School of Public Health and Health Services, Global Health<br>Khatry, Subarna; Nepal Nutr Intervent Project Sarlahi<br>Katz, Joanne; Johns Hopkins University Bloomberg School of Public Health, International Health

**Primary Subject Heading**:	Public health
Secondary Subject Heading:	Global health, Paediatrics, Research methods, Obstetrics and gynaecology
Keywords:	PUBLIC HEALTH, Maternal medicine < OBSTETRICS, NEONATOLOGY

SCHOLARONE™
Manuscripts

I, the Submitting Author has the right to grant and does grant on behalf of all authors of the Work (as defined in the below author licence), an exclusive licence and/or a non-exclusive licence for contributions from authors who are: i) UK Crown employees; ii) where BMJ has agreed a CC-BY licence shall apply, and/or iii) in accordance with the terms applicable for US Federal Government officers or employees acting as part of their official duties; on a worldwide, perpetual, irrevocable, royalty-free basis to BMJ Publishing Group Ltd ("BMJ") its licensees and where the relevant Journal is co-owned by BMJ to the co-owners of the Journal, to publish the Work in this journal and any other BMJ products and to exploit all rights, as set out in our licence.

The Submitting Author accepts and understands that any supply made under these terms is made by BMJ to the Submitting Author unless you are acting as an employee on behalf of your employer or a postgraduate student of an affiliated institution which is paying any applicable article publishing charge ("APC") for Open Access articles. Where the Submitting Author wishes to make the Work available on an Open Access basis (and intends to pay the relevant APC), the terms of reuse of such Open Access shall be governed by a Creative Commons licence – details of these licences and which Creative Commons licence will apply to this Work are set out in our licence referred to above.

Other than as permitted in any relevant BMJ Author's Self Archiving Policies, I confirm this Work has not been accepted for publication elsewhere, is not being considered for publication elsewhere and does not duplicate material already published. I confirm all authors consent to publication of this Work and authorise the granting of this licence.

For peer review only

Development of an imputation model to recalibrate birthweights measured in the early neonatal period to time at delivery and assessment of its impact on size-for-gestational age and low birthweight prevalence estimates: a secondary analysis of a pregnancy cohort in rural Nepal

Elizabeth Hazel[a], PhD, Luke C. Mullany[a], PhD, Scott L. Zeger[a], PhD, Diwakar Mohan[a], DrPH MD,Seema Subedi[a], MPH, James M. Tielsch[b], PhD MHSc, Subarna K. Khatry[a,c], MBBS, Joanne Katz[a], ScD.

Affiliations: [a]Johns Hopkins Bloomberg School of Public Health, Baltimore, Maryland, USA; [b]George Washington University Milken Institute School of Public Health, Washington DC, USA; and [c]Nepal Nutrition Intervention Project-Sarlahi (NNIPS), Sarlahi, Nepal.

Correspondence to: Elizabeth Hazel, Department of International Health, Johns Hopkins Bloomberg School of Public Health, 615 N. Wolfe St. Room W5504**,** Baltimore, MD 21205, USA, ehazel1@jhu.edu, +14106148821

For peer review only

Abstract

Objectives: In low-income countries, birthweights for home deliveries are often measured at the nadir when babies may lose up of 10% of their birthweight, biasing estimates of small-for-gestational age (SGA) and low birthweight (LBW). We aimed to develop an imputation model that predicts the "true" birthweight at time of delivery.

Design: We developed and applied a model that recalibrates weights measured in the early neonatal period to time=0 at delivery and uses those recalibrated birthweights to impute missing birthweights.

Setting: This is a secondary analysis of pregnancy cohort data from two studies in Sarlahi district, Nepal.

Participants: The participants are 457 babies with daily weights measured in the first 10 days of life from a subsample of a larger clinical trial on chlorhexidine (CHX) neonatal skin cleansing and 31,116 babies followed through the neonatal period to test the impact of neonatal massage oil type (Nepal Oil Massage Study (NOMS)).

Outcome measures: We developed an empirical Bayes model of early neonatal weight change using CHX trial longitudinal data and applied it to the NOMS dataset to recalibrate and then impute birthweight at delivery. The outcomes are size-for-gestational age and LBW.

Results: When using the imputed birthweights, the proportion of SGA is reduced from 49% (95% CI: 48-49%) to 44% (95% CI: 43-44%). Low birthweight is reduced from 30% (95% CI: 30-31%) to 27% (95% CI: 26-27%). The proportion of babies born large-for-gestational age increased from 4% (95% CI: 3.6-4.1) to 5% (95% CI: 4.5-5.0).

For peer review only - http://bmjopen.bmj.com/site/about/guidelines.xhtml

Conclusions: Using weights measured around the nadir overestimates the prevalence of SGA and LBW. Studies in low-income settings with high levels of home births should consider a similar recalibration and imputation model to generate more accurate population estimates of small and vulnerable newborns.

Strengths and limitations of this study

- A strength of this study is that we developed our model of early neonatal growth using longitudinal data of daily weight change.
- Another strength is that the data used to develop this model was sampled from a similar population of infants in rural Nepal as was the study used to apply the model; although there may be differences due to secular trends, the median birthweights and neonatal mortality rates of the two studies are similar.
- A further strength is that both studies included high quality gestational age and weight measurements taken on a population-representation sample with relatively low levels of missing data.
- A limitation of the study is that longitudinal data used to build the recalibration model did not have multiples or early neonatal deaths - babies that likely have different early growth patterns - although we are able to impute birthweight for multiples and neonatal deaths in the application model, it was based on measured weights only.
- Another limitation is that the model may not be generalizable to low-income settings outside of South Asia where prevalence of intrauterine growth restriction is lower.

For peer review only - http://bmjopen.bmj.com/site/about/guidelines.xhtml

Introduction

In 2010, 32.4 million babies in low-and-middle income countries (LMICs) were estimated to be small-for-gestational age (SGA), over a quarter of all live births and these babies had an 83% higher risk of dying the first month of life.[1,2] The South Asia region has the highest prevalence of SGA in the world; a third of babies are born small (34%) compared to the global rate (19%).[3] SGA is defined as a birthweight-for-gestational age less than the 10th percentile of a standard infant reference population and severe SGA is less that the 3rd percentile. A low weight at birth (defined as babies born less than 2500g regardless of gestational age) may result from a preterm birth (births less than 37 completed weeks gestational age), intra-uterine growth restriction (IUGR), or a combination of the two. The etiology of IUGR is likely distinct from those born preterm, and perhaps even different from babies born early with IUGR. To better understand the epidemiology of low birthweight babies, practitioners and researchers have moved to monitoring birthweight by gestational age, although low birthweight prevalence is still appropriate to use in settings where gestational age measured by ultra-sound in the first trimester (the gold standard) is rare.[4] Some babies will be on the smaller end of the curve naturally, but in LMICs where SGA estimates are high, it can be used as a proxy for IUGR prevalence. Large-for-gestational age (LGA) or babies with a birthweight >90th centile have associated health risks. [5,6] SGA, LGA and low birthweight prevalence in LMICs are essential indicators of population health.

Along with gestational age, an accurate birthweight measured close to the time of birth is critical for estimating SGA. This is because neonatal weight decreases for the first 2-3 days after birth (known as the *nadir* or period of minimum neonatal weight), before starting to increase. A systematic review of breastfed, healthy, singleton babies from high income countries found the nadir of mean weight loss of 7-8% from birthweight at day three. Many neonates in these studies

For peer review only - http://bmjopen.bmj.com/site/about/guidelines.xhtml

had weight loss greater than 7% and up to 10% from birthweight for exclusively breastfed babies.[7]

Birthweight is typically measured shortly after delivery for babies born at health facilities. For babies born outside of facilities in LMICs, it may take 24 to 72 hours before a health worker can measure weight, often at the nadir of the infant's weight loss. Weights of babies taken during the nadir may overestimate SGA and low birthweight prevalence and underestimate LGA.

In this study, we aimed to generate estimates of SGA, LGA, and low birthweight using an imputation model that extrapolates infant weights measured post-delivery (around the nadir) to a recalibrated weight at the time of birth and then uses those recalibrated birthweights to impute for babies missing a weight measurement.

Methods

Data sources

This was a secondary data analysis of two pregnancy cohort datasets from rural Nepal (table 1). The first was derived from a cluster-randomized community-based trial examining the effect of chlorhexidine full body wipe at delivery and chlorhexidine umbilical stump application on neonatal omphalitis and neonatal mortality (CHX). [8,9] From 2002 to 2005, pregnant women were recruited approximately in the sixth month of pregnancy and infants were followed-up on 1, 3, 7, 10, 14, 21 and 28 of days of age. The second was from another cluster-randomized community-based trial called the Nepal Oil Massage Study (NOMS), (clinicaltrials.gov #NCT01177111). From 2010-2017, pregnant women were enrolled through community surveillance and their newborns were randomized to receive postnatal massage using either sunflower or mustard seed oil to determine the impact of oil type on neonatal morbidity and

For peer review only - http://bmjopen.bmj.com/site/about/guidelines.xhtml

mortality. Both studies took place in Sarlahi district in southern Nepal. In this area, less than half of the women delivery in health facilities (40%), c-sections are very low (<1%) and generally 90% of infants are breastfed within the first day of delivery. [10]

Table 1: Description of data sets used for analysis

	**Training data**	**Study data**
Name	Chlorhexidine trial (CHX)	Oil massage trial (NOMS)
Setting	Sarlahi district, Nepal	Sarlahi district, Nepal
Years of data collection	2002-05	2010-17
Number of livebirths included in the analysis	457	31116
Male gender (%)	57.6<br>95% CI: 53.0 – 62.0	51.9<br>95% CI: 51.3 – 52.4
Median gestational age at delivery (weeks)	39.7<br>IQR: 37.8 – 41.2	39.4<br>IQR: 38.0 – 40.9
Median birthweight (grams)	2706<br>IQR: 2480 – 2986	2740<br>IQR: 2450 - 3040
Time of first weight measurement after birth (hours)		
Median	4.1<br>IQR: 3.1 – 5.2	15.2<br>IQR: 7.9 – 25.3
Mean	4.0<br>SD: 1.28	89.1<br>SD: 275.42
Pre-term, <37 weeks gestational age (%)	14.9<br>95% CI 11.9 – 18.5	15.7<br>95% CI: 15.3 – 16.1
Health facility delivery (%)	3.7<br>95% CI 2.3-5.9	58.1<br>95% CI 57.6-58.7
Gestational age ascertainment	Last menstrual period	Last menstrual period
Instrument used to measure infant weight	Seca Digital Scale Model 727 and Tanita digital infant weight scale	Tanita digital infant weight scale

In the CHX trial, a subset of infants (n=457) with first weight measured in the first six hours after delivery were purposively sampled for the longitudinal sub-study, and then followed for daily weight measurements through the first 10 days of life. Gestational age was calculated using last menstrual period (LMP). Multiples and babies that did not survive the early neonatal period were not included in the CHX subsample of longitudinal data. We selected this data to model early

For peer review only - http://bmjopen.bmj.com/site/about/guidelines.xhtml

neonatal weight change since it drew from the same population (but earlier timepoint) as the NOMS study.

For the NOMS study, women in the study area were visited approximately every five weeks and asked about the timing of their LMP. Pregnant women were enrolled throughout the pregnancy (median gestational age at enrollment was 14.1 weeks) and some women contributed multiple pregnancies to the study data. Birthweight was collected at the first postnatal visit on all surviving liveborn infants. The distribution of visit timing is skewed right since 8% of the babies had their first postnatal visit after day 10 (table 1). Information on maternal age, parity, education, household wealth and infant sex and survival status were collected through interviews with the mother. In both studies, birthweight was measured by trained study staff using digital scales.

Descriptive analysis

For the CHX longitudinal data, we calculated the median and interquartile range for nadir weight, time in days to reach the nadir, and time in days to return to the birthweight in the 10-day period. To make these estimates, we developed a function that extends the predicted weights from time of delivery to a maximum observed time in units of days (within the 10-day period). The function then finds the time in days when the predicted weight achieves its minimum and when the weight returns closest to the birth weight. We plotted a survival curve for probability of return to birthweight within the 10 days. Babies that did not return to their birthweight in the 10-day period were censored.

For the NOMS data, we described the sample and analyzed the pattern of missing weights and weights taken greater than 72 hours after delivery to determine if babies with missing and non-missing weights differed by preterm status (<37 weeks), survival status through the neonatal period, and mother's parity and educational level.

For peer review only - http://bmjopen.bmj.com/site/about/guidelines.xhtml

Predicting birthweight

We imputed weight at birth in the NOMS dataset by estimating, then recalibrating from the conditional distribution of each child's birthweight (weight at t=0) given a single measurement at a known later time t>0 (case 1) or imputing given missing weight (case 2).

In both cases, the strategy had three steps: (1) use the longitudinal CHX study to estimate the dependence of birthweight on later weights and assume this relationship holds approximately for the NOMS data as well; (2) use the NOMS data to model the dependence of population average weight on age at measurement and other key predictors; (3) integrate (1) and (2) to obtain the optimal linear predictor of birthweights in NOMS given the single weight measurement on each child at age t.

$$Yij = \beta_0 + \ \beta_1 X_{1ij} \ + \ \ldots \beta_p X_{pij} + \ u_{0j} + u_{ij}x_{1ij} \ + e_{ij}$$

For the first step, the CHX data was used to fit a linear mixed effect model with the repeated weights as the outcomes (Y is the daily weight for j^{th} of n_i for each baby). The fixed effects (X_{1ij}.... X_{pij}) included parity, maternal education/age, infant sex, and gestational age at birth in weeks with a spline at 40 weeks. The random effects within baby include intercept (u_{0j}) and random slope for age ($u_{ij}x_{1ij}$).

This model enabled us to estimate the conditional distribution of the unknown birthweight given one or more weights at later times, controlling for fixed effects. The mean of this distribution is the empirical Bayes estimate of birthweight. We applied the variance parameters from the training model to NOMS data to generate the distribution of the imputed birthweights for NOMS (Box 1).

For the second step, using NOMS data, we regressed the observed weight (w_t) at age t in hours on: a smooth function of maternal age and education in years, the neonate's survival status by day

For peer review only - http://bmjopen.bmj.com/site/about/guidelines.xhtml

of age, infant sex, an indicator of preterm birth, and whether the birth was a multiple or singleton, and whether it was the mother's first birth (parity) using a linear mixed effect model (Y the estimated weight for a ith woman's j[th] pregnancy that resulted in a livebirth with a measured weight). Neonatal survival status was defined as a liveborn death in the first 28 days of life with the reference being children who survived the neonatal period (28 days after birth) or alive at the

Box 1: Formulas for calculating the empirical Bayes estimate of weight and its variance, t=0

w_{it}= measured weight for child *i* at time *t>0*, NOMS

w_{it}^{NOMS} = predicted weight at actual measurement time (t>0), NOMS

w_{i0}^{NOMS} = predicted weight, t=0 from the linear mixed effect model, NOMS

G = 2x2 variance-covariance matrix of random effects in the linear mixed effects model, CHX

σ^2= residual variance from the linear mixed effects model, CHX

$Z_i = n_i \times 2$ random effects design matrix where n_i is the number of ages from which are being used to predict the birthweight, here n_i=1 and $Z_i=(1, age_i)$, NOMS

$v_i = Z_i G t(Z_i)$ = variance of the linear combination of the random effects that contribute to the birthweight prediction, NOMS, CHX

w_{i0}^{NOMS} = empirical Bayes estimate of birthweight = $w_{i0}^{NOMS} + v_i^2/(v_i + \sigma^2)(w_{it} - w_{it}^{NOMS})$

$Var(w_{i0}^{NOMS}) = (G_{11} + \sigma^2) - v_i^2/(v_i + \sigma^2)$

time they were lost-to-follow-up. We used this model to obtain the estimated weight (w_{it}^{NOMS}) for infant *i* at their measurement time *t* and their predicted birth weight (w_{i0}^{NOMS}) at *t*=0 (Box 1).

In the final step (3), we applied the relationship between birthweight and subsequent weights derived in step (1) to the NOMS observed and predicted weights to obtain an improved prediction (recalibration) of the NOMS infant's birthweights (Box 1). We generated m=5 random Gaussian variates of the imputed birthweight.

We used these recalibrated weights to impute for infants with missing weight data (case 2). We fit a random forest model to the recalibrated birthweight dataset with baby's gender, gestational age at delivery, multiple/singleton, neonatal survival status, and maternal parity, education, and age.

For peer review only - http://bmjopen.bmj.com/site/about/guidelines.xhtml

Using this model, we then imputed birthweight for babies with no weights measured and randomly generated a m=5 dataset for imputation. Infants with missing parity, survival status, maternal age/education, gestational age, singleton/multiples, or sex data were excluded from the imputation. All modelling for case 1 and case 2 were performed in R. [11]

Comparing size-for-gestational age and low birth weight using raw and imputed weights

We generated appropriate for gestational age (AGA) (10th – 90th centiles), SGA (<10th centile), and LGA (>90th centile) using Intergrowth-21 standards extrapolated up to 22+0 to 44+6 weeks GA[1] (original model was 24+0 to 42+6 weeks). [12] We generated imputed estimates of AGA, SGA, LGA and low birthweight (< 2500g) and very low birthweight (<1500g) prevalence with multiple imputation (m=5) in Stata 16.1. [13] The main outcome was the prevalence estimate comparison using the imputed birthweight versus measured weight within 72 hours. As a sensitivity analysis, we compared the proportion of term and preterm babies, female and male gender babies, and the neonatal mortality rate using the dataset with the raw birthweight versus the imputed dataset to see whether babies are being included or excluded with the imputation.

Ethical considerations

This is a secondary data analysis study of two trials for which institutional review board approvals were obtained from the Johns Hopkins Bloomberg School of Public Health (FWA00000287) and Nepal Health Research Council (FWA 00000957). All human subjects in both studies gave their informed consent to participate. This secondary data analysis was considered exempt by the Johns Hopkins Bloomberg School of Public Health institutional review board and conforms to the principles embodied in the Declaration of Helsinki.

[1] Personal Communication, Eric Ohuma, London School of Hygiene and Tropical Medicine, September 2021

For peer review only - http://bmjopen.bmj.com/site/about/guidelines.xhtml

Patient and public involvement

The patients and public were not involved in the design, conduct, reporting or dissemination of this research. Accurately measuring the health and nutritional status of infants is important to clinicians, researchers and families in Nepal, however, we did not seek the public's priorities, experience or preferences when designing this research question. We did not contact the original participants in the two studies used in this secondary data analysis.

Results

The median birthweight in the CHX trial (training data) was 2726 (IQR 2490 – 2976) (table 2). The median time of nadir was 2.1 days and the median weight at the nadir was 2609 (IQR 2370 – 2859); a relative weight loss of 4% or 117 grams. Median return to birthweight was 4.5 days and 84 infants did not regain their birthweight within the 10 days (fig. 1).

Table 2: Neonatal growth during the first 10 days of life, chlorhexidine trial (n= 4148 weight measurements on 456 infants)

	Median (IQR)
Birthweight (grams)	2726.3<br>(2490.2 – 2975.9)
Nadir weight (grams)	2609.0<br>(2369.5 – 2859.1)
Time to reach nadir (days)	2.1<br>(2.0 – 2.2)
Time to return to birthweight (days)	4.5<br>(3.8 – 5.7)
Number of babies that did not return to birthweight within the 10 days	84

IQR: Interquartile range.

From the model outputs, we found babies born to mothers with no previous births were estimated to have a lower than average birthweight and baby girls born at a greater gestational age born to older mothers were estimated to have higher birthweights on average (Annex table A1). There was

For peer review only - http://bmjopen.bmj.com/site/about/guidelines.xhtml

minimal impact of maternal education on birthweight adjusted by other covariates. We compared this model to others excluding different maternal level factors and the full model has the best fit with small differences in the Akaike's *An Information Criteria* (AIC) (Annex table A2).

There were 31,116 livebirths from 22,495 women in the NOMS dataset (table 3). We imputed a birthweight on 99% of the livebirths (n=30,996) with complete covariate and gestational age data. Of those livebirths, 3,863 were missing a weight measurement and we were able to impute a birthweight for 98% (n=3,769). The pattern of missing birthweights was not random (Annex table A3). Infants without a measured weight were more likely to be born to nulliparous mothers with more education. Infants who were preterm were more likely to have a missing birthweight (16% preterm compared to 12% full term) and over half of deceased infants did not have a weight measured (62%) compared to a tenth of infants who survived during the neonatal period (11%). Those infants with a weight measured after 72 hours showed a similar pattern (Annex table A3).

Table 3: Description of covariate data for all live births, NOMS study

	**All live births (n=31,116) % (n)**
Maternal age at LMP (years)	
<18	15.5 (4,823)
18-35	82.3 (25,622)
>35	2.2 (671)
Missing	0
Maternal education	
No school	67.0 (20,855)
1-5 years	8.5 (2,638)
>5 years	24.4 (7,595)
Missing	0.1 (28)
Parity (live or stillbirth)	
No previous births	31.5 (9,794)
1	27.4 (8,531)
2	19.6 (6,108)
3	10.8 (3,370)
4+	10.1 (3,147)

For peer review only - http://bmjopen.bmj.com/site/about/guidelines.xhtml

Missing	0.5 (166)
Gestational age	
Term (≥37 - <45 weeks)	84.3 (26,243)
Moderate-to-Late preterm (≥32-<37 weeks)	13.7 (4,262)
Early preterm (≥28-<32 weeks)	1.6 (501)
Extremely preterm (<28 weeks)	0.4 (110)
Missing	0
Infant sex	
Female	48.0 (14,925)
Male	51.7 (16,099)
Missing	0.3 (92)
Multiple births	
Singleton	98.4 (30,616)
Twin/ Triplets	1.6 (500)
Missing	0
Infant survival status	
Alive at 28 days	96.6 (30,041)
Lost-to-follow-up	3.5 (1,076)
Deceased by 28 days	3.3 (1,031)
Missing	(0.1) 44
Missing birth weight	12.4 (3,863)
Timing of birthweight measurement (Among non-missing birthweight, n=27,253)	
0-5 hours	17.5 (4,775)
6-23 hours	57.9 (15,773)
24-72 hours	11.1 (3,018)
>72 hours	13.5 (3,685)
Timing missing	2

For the NOMS imputation, at day 0 the imputed birthweights (mean 2769g, 95% CI: 2763-2775g) were on average 50g higher than the measured weights (mean 2719g, 95% CI: 2713 - 2725g) and on average 131g higher on days 1-3 (fig. 2). During days 4-9 and 10+, when the baby begins to regain and surpass birthweight, the recalibration reduces the weights back down (on average a 23g reduction for measurements taken on days 4-9 and an 857g average reduction for measurements taken on day 10 or more after delivery (fig. 2). On average, babies that did not survive the neonatal period had an imputed weight 324g less than babies that did, and multiples had an imputed weight 659g less than singleton babies (Annex Table 1). There was a small amount of heaping at 4500g

For peer review only - http://bmjopen.bmj.com/site/about/guidelines.xhtml

for the measured weights; n=70 babies at exactly 4500g or a heaping index of 35% (+/- 250 grams) (fig. 2).

Using the imputed birthweights, we found differences in the SGA prevalence compared to weights taken within 72 hours after delivery (table 4). Using the imputed birthweight decreased the proportion of babies with low birthweight from 30% (95% CI: 30-31%) to 27% (95% CI: 26-27%). SGA prevalence using measured weights was 49% (95% CI: 48-49%) and 44% (95% CI: 43-44%) using imputed weights. The prevalence of LGA babies was similar in absolute terms (4% using the raw birthweights compared to 5% using the imputation). However, in relative terms, this is a 24% increase in the estimate of LGA from the measured to imputed. The proportion of low birthweight babies decreased from 30% (95% CI: 30-31%) to 27% (95% CI: 26-27%) and the proportion of very low birthweight babies (less than 1% of the sample) remained the same.

The sensitivity analysis showed no changes in baby gender or term or preterm delivery, except for very preterm. The proportion of babies born at 28-32 weeks increased significantly (95% CI not overlapping) using the imputed dataset. Also, the neonatal mortality rate increased 109 percent from 16 deaths to 33 per 1000 livebirths (Table 4). Among the babies with non-missing birthweights, the mean birthweight measured within 72 hours and the imputed birthweight was not statistically significantly among neonatal deaths.

Table 4: **Comparison of low birthweight, small-for-gestational age, and large-for-gestational age estimates**

	Measured weight <72hr	Imputed birthweight	Relative change (%)	Absolute difference
Birthweight in grams; %, (95% CI)				
Low birthweight (<2500)	30.1 (29.5 – 30.7)	26.7 (26.0 – 27.4)	11.3	- 3.4
Very low birthweight (<1500g)	0.5 0.4 – 0.6	0.5 0.4 – 0.6	0	0
Size for gestational age in percentile; %, (95% CI)				

For peer review only - http://bmjopen.bmj.com/site/about/guidelines.xhtml

Appropriate for gestational age (90th - 10th centile)	47.7 (47.1 – 48.3)	51.6 (50.9 – 52.2)	8.2	+ 3.9
Small-for-gestational age (<10th centile)	48.5 (47.9 – 49.1)	43.7 (43.1 – 44.3)	10.0	- 4.8
Large-for-gestational age (>90th centile)	3.8 (3.6 – 4.1)	4.7 (4.5 – 5.0)	23.7	+1.0
Gestational age at delivery (weeks); % (95% CI)				
Term births (37 – 42)	74.3 73.7 - 74.9	73.3 72.8 - 73.8	1.3	-1.0
Moderate – to – late preterm (32-37)	13.4 13 - 13.8	13.7 13.3 - 14.1	2.2	+ 0.3
Very preterm (28 – 32)	1.3 1.2 - 1.4	1.6 1.5 - 1.8	23.7	+ 0.3
Extremely preterm (24 – 27)	0.3 0.2 - 0.3	0.4 0.3 - 0.4	33.3	+ 0.1
Post term (42-45)	10.8 10.4 - 11.2	11.0 10.6 - 11.3	1.9	+ 0.2
Female gender; %, (95% CI)	48.6 47.9 - 49.2	48.1 47.6 - 48.7	1.0	- 0.5
Male gender; %, (95% CI)	51.5 50.8 – 52.1	51.9 51.3 – 52.5	0.8	+ 0.4
Neonatal mortality rate; Deaths per 1000 livebirths (95% CI)	15.9 14.4 - 17.6	33.2 31.3 - 35.3	108.8	17.3
Birthweight in grams among babies with non-missing weight; mean (95% CI)				
Neonatal period survival	2713.2 2707.8 - 2718.7	2775.5 2770.0 - 2781.0	2.3	62.3
Neonatal death	2236.9 2171.8 - 2302.0	2290.0 2226.1 - 2353.9	2.4	53.1

CI: confidence intervals; pp: percentage points.

Discussion

Birthweights measured during the nadir bias estimates of SGA, LGA and low birthweight status. Our recalibration model pulled the weights back to t=0 at delivery, increasing the weights measured at the nadir, and decreasing weights measured after the nadir as the baby begins to surpass birthweight. The imputation also generated birthweight estimates for babies missing a measured weight, many of whom were early neonatal deaths and/or very preterm babies who died before the first postnatal study visit. In this dataset, these factors resulted in a reduced prevalence

For peer review only - http://bmjopen.bmj.com/site/about/guidelines.xhtml

of small, vulnerable newborns. The proportion of babies with SGA is reduced by a relative 10% and low birthweight by a relative 11% when using the recalibrated and imputed birthweights. Fonseca et al., modelled neonatal weight loss among full term, breastfed, singleton Portuguese infants with weight measured within 96 hours. [14] They fit several longitudinal models of weight change, but the goal was to examine the patterns and estimate a nadir, not to extrapolate the infant weights back to a "true" birthweight. This model was done in a high income setting with low SGA prevalence and different patterns of facility delivery/c-section compared to many LMICs in South Asia.

In the first week after delivery, there is evidence that infants follow a broadly consistent pattern of weight change. Weight loss two to three days postnatally is primarily due to loss of water weight as the cardio-pulmonary system readapts for life outside the womb. [15] Even babies born preterm lose weight primarily due to reduced extracellular water volume. [16,17] However, IUGR babies may have a different pattern of weight change. One study of SGA/term and SGA/preterm babies found weight loss was due primarily to catabolism rather than loss of extracellular volume. [18] Another study comparing SGA to AGA, preterm babies found less postnatal diuresis in the SGA babies. [19] In settings with a high prevalence of SGA, population level estimates of postnatal growth in the first week are likely to be different compared to low SGA prevalence areas – due to different growth patterns in babies with IUGR. This model should be tested in other settings but has the potential to be used for correction of birthweight measured in the days post-delivery, particularly in South Asian LMICs where SGA prevalence is high compared to other regions.

The main strength of this study is that we had a very large dataset with a relatively small proportion of missing covariates. In both the CHX and NOMS studies, we have high quality gestational age at birth and weight was measured on a population-representative sample. The two studies were

For peer review only - http://bmjopen.bmj.com/site/about/guidelines.xhtml

conducted in the same geographic area, from the same study site, and were managed by the same research organization, the Nepal Nutritional Intervention Study Sarlahi. Another strength of this study is we were able to use daily, longitudinal weights to build the model of early neonatal growth.

One limitation is the differences between the two datasets. Difference in timing of data collection: CHX (2002 – 2005) versus NOMS (2010-2017). Babies born during the NOMS data collection may have a different weight change pattern compared to babies born in CHX trial due to secular trends in maternal health and nutritional status. The percent of women delivering at home has declined from CHX to NOMS, primarily due to the cash incentive program in Nepal that pays women to deliver in a facility.[20] However, the median birthweights (table 1) and the neonatal mortality rates are similar (30 neonatal deaths per 1000 livebirths in CHX and 31 per 1000 live births[2] in NOMS). [9]

All infants in the CHX subsample of longitudinal weights survived through the neonatal period and were singleton births. This is an important difference in the underlying population of the two samples (CHX versus NOMS) since multiples typically have lower birthweight and very SGA/preterm/low birth weight babies are more likely to die in the few days after delivery. The imputed and measured mean birthweights by survival status were similar, among the babies with non-missing birthweight, indicating that the imputation model performed well (table 4).

Another limitation is that the birthweights are not missing at random in NOMS. More than half the infants died in the NOMS study before their weight could be measured, and more preterm babies did not have a weight measurement (likely also due to early neonatal death). We found a higher proportion of very preterm babies in the sample with the imputed birthweights and a much

---

[2] Personal communication, Joanne Katz, Johns Hopkins Bloomberg School of Public Health, November 2021

For peer review only - http://bmjopen.bmj.com/site/about/guidelines.xhtml

higher neonatal mortality rate (table 4). We found infants of women with more education and wealth were missing birthweight, perhaps because they were more mobile and more likely to move out of the study area, or more likely to deliver at a facility and returned home after some days. Nulliparous women were also more likely to have no weights measured on their infants. This may be due to the custom of women delivering their first baby at their parents' home. We included these covariates in the model to adjust for these differences but there is likely still residual confounding.

Conclusion

Using weights measured at or around the nadir – as typically occurs during community-based studies due to the logistics of quickly transporting data collection teams - overestimates the prevalence of SGA and low birthweight. We found a lower prevalence when using birthweight imputed to the time of delivery compared to weight taken within 72 hours post-delivery in a sample of deliveries in rural Nepal – an area where less than half of women deliver at facilities, high levels of exclusive breastfeeding and only half of babies are born with an appropriate weight for gestational age. Other studies using birthweight measurements taken from infants born at home should consider a similar imputation model to generate more accurate SGA/low birth weight status at the population level.

Overall, more research on improving estimates of SGA is needed. This model may be generalizable to other South Asian LMIC countries but may not be in LMICs with lower SGA prevalence, such as Sub-Saharan Africa. Cohort studies with birthweight measurements should consider measuring longitudinal, early neonatal weight change since data in more diverse settings is needed, especially if a similar birthweight recalibration and imputation is planned. A recalibration model may also be clinically useful to better discriminate smaller babies, improving

For peer review only - http://bmjopen.bmj.com/site/about/guidelines.xhtml

specificity of care. Finally, we note this model is restricted to livebirths since neither study measured weights on stillbirths. It would be culturally inappropriate to ask families to retain the fetal remains until study staff could arrive for weight measurement. However, IUGR may be an important cause of fetal death in this area and excluding stillbirths will likely underestimate the prevalence of SGA. Our study could not address this but including stillbirths in global estimates is critical to understanding the burden of IUGR and further work is needed to determine whether stillbirth weights may be imputed.

For peer review only

For peer review only - http://bmjopen.bmj.com/site/about/guidelines.xhtml

Contributors

Prof. Katz conceptualized and designed the study, designed the data collection protocols, developed the analytic plan and statistical models, and reviewed and revised the manuscript.

Prof. Mullany conceptualized and designed the study, designed the data collection protocols, revised data collection tools and supervised data collection, developed the analytic plan and statistical models, and reviewed and revised the manuscript.

Prof. Tielsch conceptualized and designed the study, designed the data collection protocols, and reviewed and revised the manuscript.

Dr. Khatry revised data collection tools and supervised data collection and reviewed and revised the manuscript.

Prof. Zeger developed the analytic plan and statistical models and reviewed and revised the manuscript.

Dr. Mohan, Ms. Subedi contributed significantly to the analysis and critically reviewed and revised the manuscript for important intellectual content.

Dr. Hazel developed the analytic plan and statistical models, carried out the analysis and drafted the initial manuscript.

All authors reviewed and approved the final manuscript as submitted and agree to be accountable for all aspects of the work in ensuring that questions related to the accuracy or integrity of any part of the work are appropriately investigated and resolved.

Competing interests

For peer review only

For peer review only - http://bmjopen.bmj.com/site/about/guidelines.xhtml

The authors have indicated they have no conflicts of interest, financial relationships, or competing interests relevant to this article to disclose.

Funding

This work was supported by the National Institute for Child Health and Human Development (R01HD092411 and R01HD060712), the Bill & Melinda Gates Foundation (OPP1084399), National Institutes of health (810-2054), and Cooperative Agreements HRN-A-00-97-00015-00 and GHS-A-00-03-000019-00 between Johns Hopkins University and the Office of Health and Nutrition, US Agency for International Development. Under the grant conditions of the Foundation, a Creative Commons Attribution 4.0 Generic License has already been assigned to the Author Accepted Manuscript version that might arise from this submission. The funder had no role in the study design, collection or analysis of data, writing of the report or the decision to submit the article for publication.

Acknowledgments

We would like to acknowledge Prof. Anne CC Lee at Harvard Medical School and Prof. Robert Black at Johns Hopkins Bloomberg School of Public Health for their technical contributions, and the study staff and participants at the Nepal Nutritional Intervention Project-Sarlahi site.

Data availability statement

Data are available upon reasonable request.

References

1. Lee ACC, Katz J, Blencowe H, et al. National and regional estimates of term and preterm babies born small for gestational age in 138 low-income and middle-income countries in 2010. *Lancet Glob Health.* 2013;1(1).

For peer review only

2. Katz J, Lee AC, Kozuki N, et al. Mortality risk in preterm and small-for-gestational-age infants in low-income and middle-income countries: a pooled country analysis. *Lancet*. 2013;382(9890):417-425.

3. Lee AC, Kozuki N, Cousens S, et al. Estimates of burden and consequences of infants born small for gestational age in low and middle income countries with INTERGROWTH-21st standard: analysis of CHERG datasets. *BMJ*. 2017;358:j3677.

4. Hughes MM, Black RE, Katz J. 2500-g Low Birth Weight Cutoff: History and Implications for Future Research and Policy. *Matern Child Health J*. 2017;21(2):283-289.

5. Chauhan SP, Rice MM, Grobman WA, et al. Neonatal Morbidity of Small- and Large-for-Gestational-Age Neonates Born at Term in Uncomplicated Pregnancies. *Obstet Gynecol*. 2017;130(3):511-519.

6. Mendez-Figueroa H, Truong VTT, Pedroza C, Chauhan SP. Large for Gestational Age Infants and Adverse Outcomes among Uncomplicated Pregnancies at Term. *Am J Perinatol*. 2017;34(7):655-662.

7. DiTomasso D, Cloud M. Systematic Review of Expected Weight Changes After Birth for Full-Term, Breastfed Newborns. *J Obstet Gynecol Neonatal Nurs*. 2019;48(6):593-603.

8. Mullany LC, Darmstadt GL, Khatry SK, et al. Topical applications of chlorhexidine to the umbilical cord for prevention of omphalitis and neonatal mortality in southern Nepal: a community-based, cluster-randomised trial. *The Lancet*. 2006;367(9514):910-918.

9. Tielsch JM, Darmstadt GL, Mullany LC, et al. Impact of Newborn Skin-Cleansing With Chlorhexidine on Neonatal Mortality in Southern Nepal: A Community-Based, Cluster-Randomized Trial. *Pediatrics*. 2007;119(2):e330-e340.

10. Ministry of Health, Nepal, New ERA, and ICF. Nepal Demographic and Health Survey 2016. 2017. Accessed January 13, 2020. https://dhsprogram.com/pubs/pdf/FR336/FR336.pdf.

11. R Core Team (2021). R: A language and environment for statistical computing. R Foundation for Statistical Computing, Vienna, Austria. URL https://www.R-project.org/

12. Papageorghiou AT, Kennedy SH, Salomon LJ, et al. The INTERGROWTH-21st fetal growth standards: toward the global integration of pregnancy and pediatric care. *Am J Obstet Gynecol*. 2018;218(2S):S630-S640.

13. StataCorp. Stata Statistical Software: Release 16.; 2019.

14. Fonseca MJ, Severo M, Santos AC. A new approach to estimating weight change and its reference intervals during the first 96 hours of life. *Acta Paediatr*. 2015;104(10):1028-1034.

15. Modi N, Bétrémieux P, Midgley J, Hartnoll G. Postnatal weight loss and contraction of the extracellular compartment is triggered by atrial natriuretic peptide. *Early Hum Dev*. 2000;59(3):201-208.

16. Singhi S, Sood V, Bhakoo ON, Ganguly NK, Kaur A. Composition of postnatal weight loss & subsequent weight gain in preterm infants. *Indian J Med Res*. 1995;101:157-162.

17. Bauer K, Versmold H. Postnatal weight loss in preterm neonates less than 1,500 g is due to isotonic dehydration of the extracellular volume. *Acta Paediatr Scand Suppl*. 1989;360:37-42.

For peer review only - http://bmjopen.bmj.com/site/about/guidelines.xhtml

18. vd Wagen A, Okken A, Zweens J, Zijlstra WG. Composition of postnatal weight loss and subsequent weight gain in small for dates newborn infants. *Acta Paediatr Scand*. 1985;74(1):57-61.

19. Bauer K, Cowett RM, Howard GM, vanEpp J, Oh W. Effect of intrauterine growth retardation on postnatal weight change in preterm infants. *J Pediatr*. 1993;123(2):301-306.

20. Pandey S. Women's knowledge about the conditional cash incentive program and its association with institutional delivery in Nepal. *PLOS ONE*. 2018;13(6).

For peer review only

Figure 1: Probability of return-to-birthweight and 95% confidence intervals, chlorhexidine trial

Note: Babies that did not return to their birthweight in the 10-day period were censored.

Figure 2: Comparison of measured weight and imputed weight in grams by the timing of the weight measurement in days after delivery, NOMS dataset

For peer review only

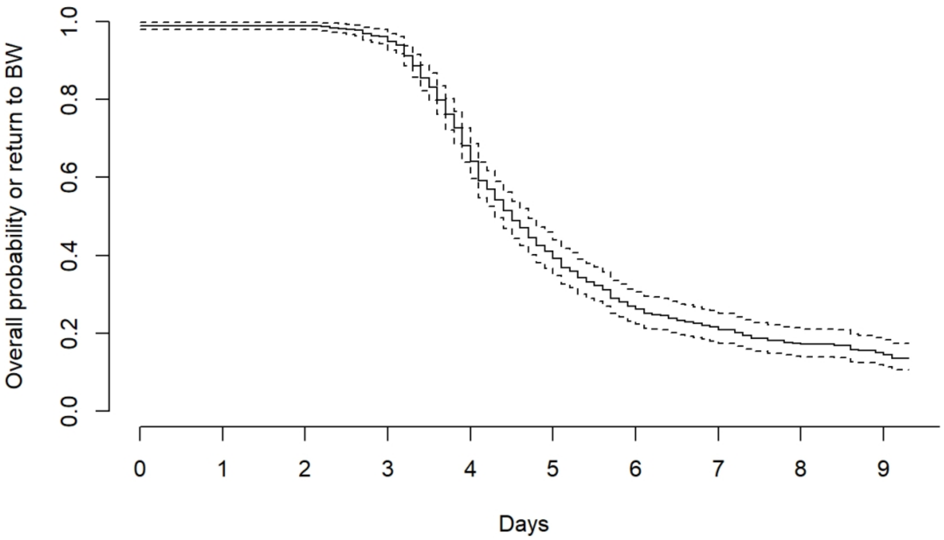

Figure 1: Probability of return-to-birthweight and 95% confidence intervals, chlorhexidine trial. Note: Babies that did not return to their birthweight in the 10-day period were censored.

467x289mm (76 x 76 DPI)

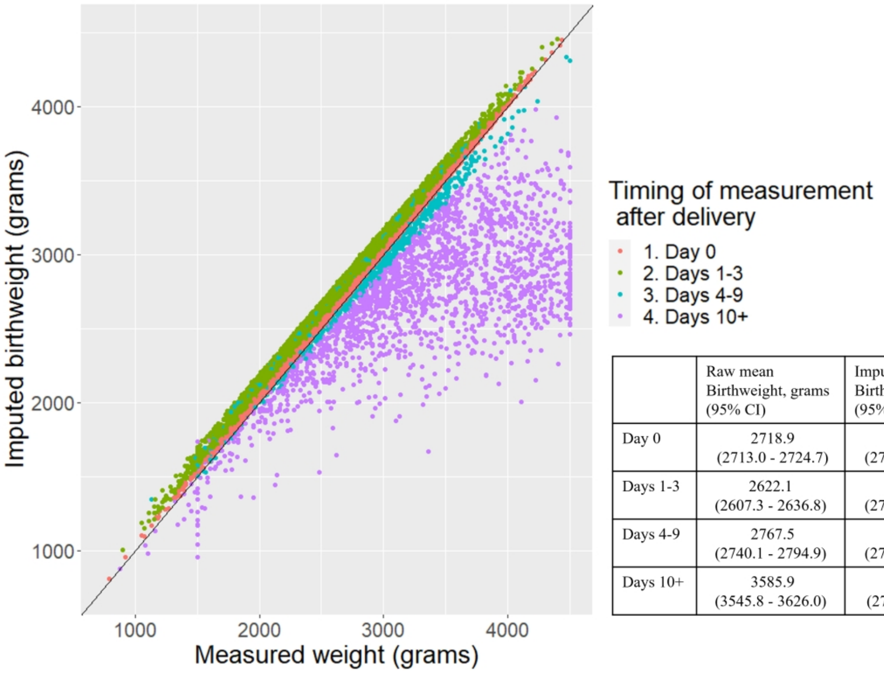

	Raw mean Birthweight, grams (95% CI)	Imputed mean Birthweight, grams (95% CI)
Day 0	2718.9 (2713.0 - 2724.7)	2768.6 (2762.7 - 2774.5)
Days 1-3	2622.1 (2607.3 - 2636.8)	2753.1 (2738.0 - 2768.2)
Days 4-9	2767.5 (2740.1 - 2794.9)	2744.7 (2716.2 - 2773.3)
Days 10+	3585.9 (3545.8 - 3626.0)	2729.4 (2700.8 - 2758.1)

Figure 2: Comparison of measured weight and imputed weight in grams by the timing of the weight measurement in days after delivery, NOMS dataset.

674x455mm (130 x 130 DPI)

Annexes:

Table A1: Regression outputs for the training model, chlorhexidine trial substudy (n= 4148 weight measurements on 456 infants) and the analytical model, NOMS trial (n=28016 infants, 27836 pregnancies and 21770 women) with complete data on the covariates.

	CHX Weight, grams (standard error)	*NOMS Weight, grams (standard error)*
Parity (reference=previous birth)	-163.7 (49.6)	-202.4<br>6.0
Sex (reference=female)	50.6 (37.7)	110.3<br>4.7
Gestational age in weeks, centered at 40 weeks	52.7 (7.9)	31.5<br>0.8
Neonatal death (reference = survives neonatal period or lost-to-follow-up)	N/A	-323.9<br>19.8
Maternal education in years	-0.5 (3.3)	13.8<br>0.7
Maternal age in years	10.7 (6.0)	5.4<br>0.6
Multiple gestation (twins or triplets) (reference = singleton)	N/A	-658.5<br>23.8
Intercept	2745.1 (90.0)	

N/A: there were no twins/triplets or neonatal deaths in the first 10 days in the CHX substudy.

Table A2: Akaike's *An Information Criteria* (AIC) for five models of predicted weight at delivery (training model), chlorhexidine trial (n= 4148 weight measurements on 456 infants)

Model	Covariates	Degrees freedom	AIC
Full model	Baby gender, Gestational age at delivery, Parity, Maternal age, and Maternal years of education,	13	48369.92
Model 1	Excluding Parity	12	48388.18
Model 2	Exclude Education	12	48376.52
Model 3	Exclude Education and Maternal age	11	48378.86
Model 4	Exclude: Education, Maternal age, and Parity	10	48396.61

AIC: Akaike's *An Information Criteria*

For peer review only - http://bmjopen.bmj.com/site/about/guidelines.xhtml

Table A3: Proportion of infants with weight missing or weight measured greater than 72 hours post-delivery stratified by preterm birth, survival status, and maternal parity and education, NOMS data

	Type of missingness % (95% CI)	
**Proportion missing with:**	**Weight missing**	**Weight measured greater than 72 hours after delivery**
Preterm birth (<37 weeks)	16.3 (15.3 – 17.4)	13.6 (12.6 – 14.7)
Non-preterm birth (>=37 weeks)	11.7 (11.3 – 12.1)	13.5 (13.1 – 14.0)
Infant alive at end of study	10.6 (10.3 – 11.0)	13.6 (13.2 – 14.1)
Infant deceased at end of study	61.7 (58.7 – 64.6)	4.8 (3.1 – 7.4)
No previous births	15.7 (15.0 – 16.4)	17.0 (16.2 – 17.8)
Any previous births	10.9 (10.5 – 11.3)	12.0 (11.5 – 12.5)
No education	11.2 (10.8 – 11.7)	11.6 (11.2 – 12.1)
Any education	14.9 (14.2 – 15.6)	17.6 16.8 – 18.4)

CI: confidence intervals

For peer review only

An imputation model to recalibrate birthweights measured in the early neonatal period to time at delivery its impact on size-for-gestational age and low birthweight prevalence estimates: a secondary analysis of a pregnancy cohort in rural Nepal.

Elizabeth Hazel[a], PhD, Luke C. Mullany[a], PhD, Scott L. Zeger[a], PhD, Diwakar Mohan[a], DrPH MD,Seema Subedi[a], MPH, James M. Tielsch[b], PhD MHSc, Subarna K. Khatry[a,c], MBBS, Joanne Katz[a], ScD.

STROBE Statement—Checklist of items that should be included in reports of ***cohort studies***

	Item No	Recommendation	Line number CLEAN version
**Title and abstract**	1	(*a*) Indicate the study's design with a commonly used term in the title or the abstract	1-3
		(*b*) Provide in the abstract an informative and balanced summary of what was done and what was found	22-52
**Introduction**			
Background/rationale	2	Explain the scientific background and rationale for the investigation being reported	69-95
Objectives	3	State specific objectives, including any prespecified hypotheses	96-99
**Methods**			
Study design	4	Present key elements of study design early in the paper	102
Setting	5	Describe the setting, locations, and relevant dates, including periods of recruitment, exposure, follow-up, and data collection	103 – 130, table 1
Participants	6	(*a*) Give the eligibility criteria, and the sources and methods of selection of participants. Describe methods of follow-up	103 – 130
		(*b*) For matched studies, give matching criteria and number of exposed and unexposed	Not applicable
Variables	7	Clearly define all outcomes, exposures, predictors, potential confounders, and effect modifiers. Give diagnostic criteria, if applicable	Primary and secondary outcomes: 184 – 192<br><br>Covariates: 155-170
Data sources/ measurement	8*	For each variable of interest, give sources of data and details of methods of assessment (measurement). Describe comparability of assessment methods if there is more than one group	103 – 130 Comparability table 1and Annex table 1
Bias	9	Describe any efforts to address potential sources of bias	Not applicable
Study size	10	Explain how the study size was arrived at	Available at citations of original studies
Quantitative variables	11	Explain how quantitative variables were handled in the analyses. If applicable, describe which groupings were chosen and why	155-170
Statistical methods	12	(*a*) Describe all statistical methods, including those used to control for confounding	144-182
		(*b*) Describe any methods used to examine subgroups and interactions	189-192

For peer review only - http://bmjopen.bmj.com/site/about/guidelines.xhtml

		(*c*) Explain how missing data were addressed	180-182
		(*d*) If applicable, explain how loss to follow-up was addressed	Not applicable
		(*e*) Describe any sensitivity analyses	189-192
**Results**			
Participants	13*	(a) Report numbers of individuals at each stage of study—eg numbers potentially eligible, examined for eligibility, confirmed eligible, included in the study, completing follow-up, and analysed	Available at citations of original studies
		(b) Give reasons for non-participation at each stage	Available at citations of original studies
		(c) Consider use of a flow diagram	Available at citations of original studies
Descriptive data	14*	(a) Give characteristics of study participants (eg demographic, clinical, social) and information on exposures and potential confounders	Available at citations of original studies
		(b) Indicate number of participants with missing data for each variable of interest	Available at citations of original studies
		(c) Summarise follow-up time (eg, average and total amount)	Available at citations of original studies
Outcome data	15*	Report numbers of outcome events or summary measures over time	Available at citations of original studies
Main results	16	(*a*) Give unadjusted estimates and, if applicable, confounder-adjusted estimates and their precision (eg, 95% confidence interval). Make clear which confounders were adjusted for and why they were included	232-250
		(*b*) Report category boundaries when continuous variables were categorized	Table 4
		(*c*) If relevant, consider translating estimates of relative risk into absolute risk for a meaningful time period	Not applicable
Other analyses	17	Report other analyses done—eg analyses of subgroups and interactions, and sensitivity analyses	251-256
**Discussion**			
Key results	18	Summarise key results with reference to study objectives	261-263
Limitations	19	Discuss limitations of the study, taking into account sources of potential bias or imprecision. Discuss both direction and magnitude of any potential bias	289-313
Interpretation	20	Give a cautious overall interpretation of results considering objectives, limitations, multiplicity of analyses, results from similar studies, and other relevant evidence	315-321
Generalisability	21	Discuss the generalisability (external validity) of the study results	324-328
**Other information**			
Funding	22	Give the source of funding and the role of the funders for the present study and, if applicable, for the original study on which the present article is based	328-366

*Give information separately for exposed and unexposed groups.

For peer review only

For peer review only - http://bmjopen.bmj.com/site/about/guidelines.xhtml

Note: An Explanation and Elaboration article discusses each checklist item and gives methodological background and published examples of transparent reporting. The STROBE checklist is best used in conjunction with this article (freely available on the Web sites of PLoS Medicine at http://www.plosmedicine.org/, Annals of Internal Medicine at http://www.annals.org/, and Epidemiology at http://www.epidem.com/). Information on the STROBE Initiative is available at http://www.strobe-statement.org.

For peer review only
